# Protein sorting into protein bodies during barley endosperm development is putatively regulated by cytoskeleton members, MVBs and the HvSNF7s

**DOI:** 10.1038/s41598-020-58740-x

**Published:** 2020-02-05

**Authors:** Valentin Roustan, Julia Hilscher, Marieluise Weidinger, Siegfried Reipert, Azita Shabrangy, Claudia Gebert, Bianca Dietrich, Georgi Dermendjiev, Madeleine Schnurer, Pierre-Jean Roustan, Eva Stoger, Verena Ibl

**Affiliations:** 1 Department of Ecogenomics and Systems Biology, University of Vienna, Althanstr. 14, 1090 Vienna, Austria; 2 Department for Applied Genetics and Cell Biology, University of Natural Resources and Life Sciences, Muthgasse 18, 1190 Vienna, Austria; 3 Core Facility Cell Imaging and Ultrastructure Research, University of Vienna, Althanstr. 14, 1090 Vienna, Austria; 4 Present Address: Medical University of Vienna, Department of Obstetrics and Gynecology Reproductive Biology Unit, Währinger Gürtel 18-20, 5Q, A-1090 Vienna, Austria

**Keywords:** Protein transport, Proteomics

## Abstract

Cereal endosperm is a short-lived tissue adapted for nutrient storage, containing specialized organelles, such as protein bodies (PBs) and protein storage vacuoles (PSVs), for the accumulation of storage proteins. During development, protein trafficking and storage require an extensive reorganization of the endomembrane system. Consequently, endomembrane-modifying proteins will influence the final grain quality and yield. However, little is known about the molecular mechanism underlying endomembrane system remodeling during barley grain development. By using label-free quantitative proteomics profiling, we quantified 1,822 proteins across developing barley grains. Based on proteome annotation and a homology search, 94 proteins associated with the endomembrane system were identified that exhibited significant changes in abundance during grain development. Clustering analysis allowed characterization of three different development phases; notably, integration of proteomics data with *in situ* subcellular microscopic analyses showed a high abundance of cytoskeleton proteins associated with acidified PBs at the early development stages. Moreover, endosomal sorting complex required for transport (ESCRT)-related proteins and their transcripts are most abundant at early and mid-development. Specifically, multivesicular bodies (MVBs), and the ESCRT-III HvSNF7 proteins are associated with PBs during barley endosperm development. Together our data identified promising targets to be genetically engineered to modulate seed storage protein accumulation that have a growing role in health and nutritional issues.

## Introduction

After differentiation, fully developed cereal endosperm makes up to 75% of the grain weight and covers four major cell types: aleurone, starchy endosperm, transfer cells, and the cells of the embryo surrounding region^1^. The starchy endosperm thereby is characterized as a storage site, accumulating starch and seed storage proteins (SSPs)^2^. The aleurone layer plays essential roles during seed germination and mobilizes starch and SSP reserves in the starchy endosperm by releasing hydrolytic enzymes that are responsible for the degradation of stored nutrients in the endosperm^2^. Contrary to the persistent endosperm of cereals, the cellular endosperm of *Arabidopsis thaliana* (*A. thaliana)* supports the developing and growing embryo, resulting in a gradually depleted endosperm as the embryo grows. Finally, the massive *A. thaliana* embryo is only accompanied by a single peripheral layer, the aleurone layer, in mature seeds^2^. Consequently, *A. thaliana* cannot to be used as a model system to study the endomembrane system in grain endosperm.

In cereals, SSPs, which account for more than 50% of the grain protein content^3,4^, accumulate in the outer layer of the endosperm, in the subaleurone, and in the starchy endosperm, the latter in parallel with starch granules^5^. In barley, for instance, globulins and prolamins comprise the major endosperm SSPs^6^.

The SSP trafficking routes depend on the cereal species, endosperm layer and development stage^7–9^. SSPs are produced by the secretory pathway and reach their final destinations by two main routes: soluble albumins and globulins pass the endoplasmic reticulum (ER) and Golgi to travel to protein storage vacuoles (PSVs); and most prolamins are finally deposited in specific, ER-derived protein bodies (PBs) or/and in PSVs. Along with these main routes, other organelles are proposed to be involved in the SSP trafficking in cereal endosperm, e.g., multivesicular bodies (MVBs), precursor-accumulating (PAC) vesicles and/or dense vesicles (DVs)^9^.

Depending on the protein trafficking, PB composition is affected, thereby influencing the qualitative output. For instance, the SSP composition defines the malting purposes, most specifically for the brewing industry^10^. Thus, endomembrane-modifying proteins within the endomembrane system will have an influence on the final grain quality/yield and recombinant protein production.

Barley *(Hordeum vulgare)* holds the fourth-most important cereal in terms of food production after maize, wheat, and rice^11^. Microscopic analyses revealed that spherical PSVs, which are stable in the aleurone during development, underwent a dynamic rearrangement including fusion, rupture and degeneration in the subaleurone and starchy endosperm of barley^12–14^. Between 8 and 12 days after pollination (DAP), PSVs reduced their size and even degenerated in the starchy endosperm^13^. Simultaneously, PBs were found in distinct compartments in the subaleurone but also within the vacuoles, where fusion events of PBs were observed by live cell imaging^13^. In the starchy endosperm, PBs were tightly enclosed by vacuoles that finally degenerated and released the PBs^13^. Recent bioinformatic, proteomic and RT-qPCR analyses shed the first light on proteins possibly involved in the rearrangement of the endomembrane system in barley endosperm^12,15,16^: Endosomal sorting complexes required for transport (ESCRT)-III proteins were identified by a bioinformatic assay^12^ and localization studies of recombinantly expressed ESCRT-III components HvSNF7a, HvVPS24 and HvVPS60a in barley revealed different localization of these proteins within the endosperm^12^. It was further suggested that cell layer–specific protein deposition or trafficking and remodeling of the endomembrane system in the endosperm has an impact on the steady-state association of ESCRT-III^12^. Additionally, the ER was identified to be most abundant in the starchy endosperm and ER rearrangements were characterized, including re-localization of HvPDIL1-1 during development^15^.

This work aimed to temporally map *in situ* the endomembrane system during barley endosperm development, using integrative cell biology experimental approaches. Label-free proteomics approaches of four different stages of grain development allowed the quantification of 1,822 proteins. Among these, 94 proteins could be associated with the endomembrane system. We identified cytoskeleton members, ESCRT proteins, and MVBs as putative key players for protein sorting into PBs during barley endosperm development. More specifically, seven out of eight proteins related to the ESCRT machinery showed specific expression patterns and transcript abundances associated with early and mid-development. In this context, confocal and transmission electron microscopy analyses located HvSNF7 and MVBs at the periphery of PBs and later within PBs, playing a putative role in protein sorting to PBs at mid-development. These results pave the way to exploit specifically the function of the endomembrane system to modulate SSP accumulation and/or to improve the production of recombinant proteins.

## Materials and Methods

### Plant material and growth conditions

Barley (*H. vulgare L*.) wild-type variety Golden Promise (GP) and transgenic lines (TIP (Tonoplast intrinsic protein)3-GFP, p6U::SNF7.1-mEosFP) were cultivated as described in^17^ and in the supplementary material. In detail, caryopses were harvested at different stages of grain development including 6–8, 10, 12–18 and ≥20 days after pollination (DAP) (designated as 6, 10, 12 and ≥20 DAP) of three biological replicates.

### Data processing and protein identification

Sample preparation for proteomics analyses and Mass spectrometry (MS) was performed as previously described in^16^ and in the supplementary material. Raw files were processed with MaxQuant 1.5 (http://www.maxquant.org) and the Andromeda search algorithm^18–20^ on the barley UniProt database (http://uniprot.org). Peptide identification was performed as previously described^16^. Label-free quantification was done at the MS1 level with at least two peptides per protein. PTXQC was used to assess data quality and statistical analysis was performed with Perseus 1.5 software^21,22^. Protein annotation was performed with the MERCATOR tool (http://mapman.gabipd.org/de)^23^. Unknown proteins were identified by using BLAST at the UniProt homepage searching for the most identical cereal protein. Proteins were classified to “compartment-specific proteins” (functional associated with a specific subcellular endomembrane pathway or organelle), and as “trafficking regulators” (functional associated with several organelles) based on published data. In the final dataset, representative proteins were quantified in at least 9 of the 12 samples analyzed. Data were first Log2 transformed prior Z-scores (zero mean, unit variance) and were finally used to calculate the relative protein abundance. A one-way analysis of variance (ANOVA) and Student’s t-tests were performed with Perseus 1.5 software. Cluster analysis was performed with fuzzy-c means algorithm implemented in GProX^24^. Generation of protein-protein interaction (PPI) networks was conducted via the Search Tool for the Retrieval of Interacting Genes/Proteins (STRING) database for known and predicted protein-protein interactions (http://string-db.org/) with default parameters^25^. The MS proteomic data have been deposited to the ProteomeXchange Consortium^26^ via the PRIDE (Vizcano *et al*., 2016) partner repository with the dataset identifier PXD009722.

### Cloning of constructs

#### Bimolecular Fluorescence Complementation (BiFC)

The backbone of all vectors (MKK4_SPYCE and MPK3_SPYNE, kindly provided by Dr. Andrea Pitzschke) used in this study contain a p35S promoter (Cauliflower Mosaic virus 35 S promoter), 5′UTR (untranslated region from tobacco etch virus), our genes of interest (*HvSNF7.1*), C-terminal or N-terminal sequence of YFP (SPYCE or SPYNE, t35 (Cauliflower Mosaic virus 35S terminator), HA-tag (Human influenza hemagglutinin) or c-MYC-tag, and a kanamycin antibiotic resistance sequence. *HvSNF7.1* (according to *HvSNF7a.1* described in^12^) was cloned into the vector pCR2.1 (#K200001, Thermo Fisher Scientific, Massachusetts, USA) using HvSNF7.1_NcoI-F and HvSNF7.1_NotI-R as primers, digested by NcoI and NotI and inserted into previously digested MKK4_SPYCE and MPK3_SPYNE, respectively. To obtain a pSPYCE vector without insert for control reactions, plasmids were cut (NcoI/NotI), blunted using Klenow fragment and re-ligated. All the clones were verified by sequencing.

#### Yeast two-hybrid (Y2H)

The restriction sites NdeI and Sfil were introduced into *HvSNF7.1* by PCR using the primers pGADT7_SNF7F and pGADT7_SNF7R3. Using NdeI and Sfil, *HvSNF7.1* was ligated into the vector pGEM-T Easy (#A1360, Promega, Madison, Wisconsin, United States). All the clones were verified by sequencing and finally cloned into the target vectors pGADT7 AD (#630442, Clontech Laboratories, Mountain View, California, United States) and pGBKT7 DNA-BD (#630443, Clontech Laboratories, Mountain View, California, United States). Positive clones were verified by sequencing.

#### p6U::SNF7.1-mEosFP

To obtain HvSNF7-mEosFP driven by the barley hordein D promoter (p6U) and ended by the nopaline synthase terminator, *HvSNF7.1-mEosFP* (previously described in^12^) was ligated in the following into the vector p6U_pHordeinD (kindly provided by Eszter Kapusi, unpublished) using MluI. The p6U vector backbone originates from DNA Cloning Service e.K. (http://www.dna-cloning.com/vectors/Binaries_hpt_plant_selection_marker/p6U.gbk). The sequence of the Hordein D promoter was taken from^27^. The vector p6U_pHordeinD is based on p6U_pHordein_BamHI_SP, which was cut using BamHI/HindIII, blunted using Klenow fragment and re-ligated.

### Transformation of barley endosperm cells

Barley (GP) transformation was carried out using particle bombardment^17,28^. T1 plants surviving hygromycin selection were genotyped using primer pairs as described in Supplemental Table 1. Homozygous T4 grains were used from transgenic p6U::SNF7.1-mEosFP plants for microscopic and Western blot analyses. The intact fusion protein SNF7.1-mEosFP was detected by Western blot using polyclonal rabbit anti-SNF7 antibody (kindly provided by D. Teis), which could detect all isoforms of HvSNF7 and the transgenic SNF7.1-mEosFP (49 kDa) (Supplementary Fig. 1).

**Table 1:** Identified proteins classified corresponding to their involvement within the endomembrane pathway and to their diverse endomembrane functions.

Nr.	Uniprot Accession	Protein Name	Pathway
1	M0XYS5	Coatomer subunit delta (RET2p) COPI	secretory pathway
2	M0UY14	Golgin candidate 5	secretory pathway
3	A0A287KUM9	Coatomer subunit beta (COPI)	secretory pathway
4	F2E4V3	Coatomer subunit epsilon	secretory pathway
5	A0A287HD61	SEC. 31 homolog B (COPII)	secretory pathway
6	F2CXJ0	Endoplasmic reticulum vesicle transporter protein	secretory pathway
7	A0A287HI31	Transmembrane emp24 domain-containing protein 10	secretory pathway
8	F2CQI5	Protein transport protein Sec. 61 subunit beta	secretory pathway
9	A0A287T0X1	Putative ADP-ribosylation factor GTPase-activating protein AGD8 (COPI)	secretory pathway
10	A0A287NDD5	SEC. 24 like (COPII)	secretory pathway
11	A0A287N3A5	CASP	secretory pathway
12	F2DJ14	SEC. 13 homolog B (COPII)	secretory pathway
13	F2DF14	Signal recognition particle subunit SRP72	secretory pathway
14	A0A287J9F8	Gamma-soluble NSF attachment protein	secretory pathway
15	F2CRB3	Ras-related protein RIC1 - ARA5	secretory pathway
16	A0A287WFD7	Peroxisome biogenesis protein 5 PEX5	peroxisome
17	A0A287QYS2	Proton pump-interactor 1	PM
18	M0UEQ. 6	Nicastrin	PM
19	F2CWF3	Putative voltage-gated potassium channel subunit beta	PM
20	A0A287RSX4	Proton pump-interactor 1	PM
21	F2CS48	redox.ascorbate and glutathione; Membrane steroid-binding protein 1	PM
22	A0A287QB60	SEC. 1 family transport protein SLY1	sorting
23	A0A287XZU3	CLC2 (CCV)	sorting
24	A0A287JMQ9	CLC1	sorting
25	A0A287Y199	EHS (TPLATE)	sorting
26	F2D106	VPS20.1	sorting
27	A0A287NWK0	TOL3	sorting
28	M0X0B4	TOL2	sorting
29	M0XC79	TOL1	sorting
30	A0A287X2J4	TOL8	sorting
31	A0A287R3U8	CHC1	sorting
32	A0A287FF51	SH3PH	sorting
33	M0YLE4	non-specific serine/threonine protein kinase	sorting
34	A0A287QP21	Auxilin-related protein 1	sorting
35	A0A287FUM0	SNX2b	sorting
36	A0A287U4A9	VPS29	sorting
37	A0A287K2S5	SKD1	sorting
38	A0A287H7X6	VSR1	sorting
39	A0A287NZS5	VSR1	sorting
40	A0A287R1U7	VSR1	sorting
41	F2DS44	SNX1	sorting
42	A0A287R803	SNF7.1	sorting
43	A0A287XAB9	SNF7.2	sorting
44	A0A287RZ89	PUX 8.1	transport
45	A0A287WLG4	PUX 8.2	transport
46	A0A287G9M8	Patellin1	transport
47	A5CFY5	Tubulin beta chain	transport
48	A5CFY9	Tubulin beta chain	transport
49	A0A287FFF9	Actin-2	transport
50	F2DY31	Actin-depolymerization factor 4	transport
51	A0A287MS88	Myosin-like protein	transport
52	M0YZY8	Autophagy-related protein 8 C	degradation
53	A0A287FQD8	Autophagy-related protein 3	degradation
54	A0A287UDR1	Vacuolar processing enzyme 1	vacuolar processing
55	A0A287IXX5	Vacuolar processing enzyme 2b	vacuolar processing
56	A0A287IXX4	Vacuolar processing enzyme 2c	vacuolar processing
57	A0A287IXM3	Vacuolar processing enzyme 2d	vacuolar processing
58	A0A287RKR9	Vacuolar processing enzyme 4	vacuolar processing
59	A0A287IY00	Legumain	vacuolar processing
60	A0A287GK50	Dynamin-related protein 1 A	dynamins
61	A0A287N3M7	Dynamin-related protein 1 C, putative	dynamins
62	A0A287W654	Dynamin-2A	dynamins
63	A0A287MCV3	Dynamin-related protein 3 A	dynamins
64	A0A287GAT9	NSF	SNARE
65	A0A287GJC4	SYP71 protein	SNARE
66	A0A287N705	ERO1	Disulfide-generating enzyme and- carrier
67	A0A287EWS7	HvPDIL2-1	Disulfide-generating enzyme and- carrier
68	A0A287NWD9	HvPDIL1-1	Disulfide-generating enzyme and- carrier
69	A0A287RLW1	HvPDIL2-2	Disulfide-generating enzyme and- carrier
70	A0A287P669	HvPDIL5-1	Disulfide-generating enzyme and- carrier
71	A0A287T503	HvPDIL1-3	Disulfide-generating enzyme and- carrier
72	M0XFC8	Vacuolar proton-ATPase subunit A	ATPase
73	F2DCK0	V-type proton ATPase subunit B 1	ATPase
74	A0A287L8C5	YLP; Vacuolar ATP synthase subunit E	ATPase
75	F2EFW5	Pyrophosphate-energized vacuolar membrane proton pump	ATPase
76	A0A287X931	RABA1d/d	GTPase
77	A0A287EG08	AtRABD1	GTPase
78	A0A287K336	RABD2a	GTPase
79	A0A287GB98	RABG3f	GTPase
80	A0A287HZ99	Ras-related protein RABH1b	GTPase
81	A0A287WHY3	Signal recognition particle receptor beta subunit	GTPase
82	A0A287HAL5	Signal recognition particle 54 kDa protein	GTPase
83	A0A287UM33	ADP-ribosylation factor GTPase-activating protein AGD12	GTPase-activating protein
84	A0A287FZ46	GDI1/2	RAB regulator
85	F2CQ27	GTP-binding nuclear protein	GTP binding protein
86	A0A287P5H8	Ran-binding protein	GTP binding protein
87	M0ZCE0	Ran-binding protein 1	GTP binding protein
88	M0YLZ9	Ran-specific GTPase-activating protein 2	GTP binding protein
89	A0A287QN80	GTPase SAR1A	GTP binding protein
90	M0X1Z2	Ran GTPase activating protein	GTP binding protein
91	F2CWF2	GTP-binding protein SAR1A	GTP binding protein
92	A0A287H404	GTP-binding protein	GTP binding protein
93	F2DYD4	GTP-binding protein SAR1A	GTP binding protein
94	M0YT49	ADP-ribosylation factor homolog1	GTP binding protein

### Microscopy

#### Live cell imaging

GP and the transgenic line TIP3-GFP^13^ were used for live cell imaging as previously described^13^. ER-Tracker Green (BODIPY FL Glibenclamide; #E34251, Thermo Fisher Scientific, Waltham, Massachusetts, USA) and LysoTracker Red DND-99 (#L7528, Thermo Fisher Scientific, Waltham, Massachusetts, USA) were used to visualize ER and acidic compartments, respectively. ER-Tracker Green was used as previously described^13^. In detail, at least three randomly selected transgenic and GP grains were harvested at 6, 12 and ≥20 DAP, sectioned, washed, and stained as follows: ER-Tracker Green, 1 h (final concentration, 2 µM, from 1 mM DMSO stock with water); LysoTracker Red, 30 min (final concentration, 2 µM, from 1 mM DMSO stock with water). Mock treatments included the final DMSO concentration in water. Sections were mounted in tap water and immediately imaged by the Leica SP5 CLSM using sequential scans with filter settings for GFP (excitation 488 nm, emission 500–530 nm), LysoTracker Red (excitation 561 nm, emission 570–630 nm), and ER-Tracker Green (excitation 488 nm, emission 500–531 nm). Transgenic p6U::SNF7.1-mEosFP grains were chipped, mounted in tap water and analyzed by CLSM with excitation at 488 nm, emission 508–540 nm.

#### Bimolecular Fluorescence Complementation (BiFC)

A single colony of transformed *Agrobacterium tumefaciens* was inoculated in 5 mL of YEB-Medium (0.5% beef extract, 0.5% sucrose, 0.1% yeast extract, 0.05% MgSO_4_*7H_2_O) containing appropriate antibiotics and incubated at 28 °C overnight. In the morning, 5 ml of the same medium was re-inoculated with 1 ml of the pre-culture. Cells were collected by centrifugation at 5000 *g* for 5 min and the pellet was washed with 1 mL infiltration buffer (10 mM MES pH 5.7, 10 mM MgCl_2_, 100 µM acetosyringone). Washed cells were re-collected by centrifugation (5000 *g*, 5 min) and washed two additional times with 500 µl infiltration buffer and finally adjusted to an OD_600_ of 0.3. The resuspended bacteria containing the corresponding binary expression vectors for BiFC were mixed in a ratio of 1:1 and incubated for 3 h in darkness. *Nicotiana benthamiana* plants were cultivated in the greenhouse on soil, maintained at 60% humidity, with a 14 h light period and a 25 °C day/19 °C night temperature cycle. The entire leaf area of two leaves per *Nicotiana benthamiana* plant was infiltrated with the bacterial solution through the abaxial side using a 1 mL syringe. After infiltration, the plants were kept in a tray with a hood at 25 °C. After 2–5 days, the detection of protein–protein interaction by BiFC was performed using confocal microscopy (Leica SP5 CLSM). The excitation wavelength was 514 nm (argon laser) and emission was detected between 525–600 nm and 680–760 nm for YFP and autofluorescence detection, respectively. Red channels were visualized in magenta.

#### Histological and immunofluorescence studies

At least three randomly selected GP grains were harvested at 6, 12 and ≥20 DAP and fixed, embedded and sectioned as described in^15,16^. The 1.5 µm sections on glass slides were stained with toluidine blue (0.1%) for 30 s at 80 °C on a hot plate and rinsed with distilled water. Immunofluorescence microscopy of developing barley grains was performed as described by^16^ using: polyclonal rabbit anti-V-ATPase antibody (#AS 07 213, Agrisera, Vännäs, Sweden, raised against Arabidopsis thaliana At4g11150, specific for higher plants including *Hordeum vulgare*), dilution 1:100; polyclonal rabbit anti-actin antibody (#AS 13 2640, Agrisera, Vännäs, Sweden, raised against *Arabidopsis thaliana* actin-1/-2/-3/-4/-5/-7/-8/-11 and-12, specific for higher plants including *Hordeum vulgare*), dilution 1:50; polyclonal rabbit anti-tubulin-α antibody (#AS 10 680, Agrisera, Vännäs, Sweden, raised against *Arabidopsis thaliana* tubulin alpha-1/-2/-4/-5/-6-chain, specific for higher plants including *Hordeum vulgare*, dilution 1:50); polyclonal rabbit anti-VSR1 antibody, dilution 1:100, kindly provided by Dr. Liwen Jiang, raised against *Pisum sativum* BP80, specific for *Pisum sativum*, *Arabidopsis thaliana* and BY2-cells) and polyclonal rabbit anti-SNF7 antibody (dilution 1:100, kindly provided by D. Teis, raised against *Saccharomyces cerevisiae* SNF7, specifically recognizing SNF7^29^). Goat Anti-Rabbit IgG (H + L) Cross-Adsorbed Secondary Antibody, Alexa Fluor 488 (#A-11008, Thermo Fisher Scientific, Waltham, Massachusetts, USA) (dilution 1:30) was used as a secondary antibody. The specificity of the first antibodies for GP was proved by western blot analyses (Supplementary Fig. 1.) At least three sections were analyzed, and pictures captured by Nikon Eclipse Ni. Images were processed using Leica confocal software version 2.63, ImageJ and Adobe Photoshop CS5. Negative controls show sections incubated only with the secondary antibody.

#### Transmission electron microscopy (TEM)

The coat was removed from grains harvested at 6, 12 and ≥20 DAP, chopped and immediately fixed in 4% (w/v) paraformaldehyde plus 2.5% (v/v) glutaraldehyde in 0.1 M sodium cacodylate, pH 7.4, overnight at 4 °C. After washing with sodium cacodylate buffer, the samples were immersed in OsO_4_ for one and a half hours at room temperature followed by washing with buffer. The chemically fixed samples underwent dehydration in an ethanol series (30%, 50%,70%, 95% ethanol for 10 min each, and two times 100% ethanol). Prior to infiltration with resin, the ethanol was replaced by 100% acetone. The dehydrated samples were infiltrated with low viscosity resin (Agar Scientific Ltd, Stansted, UK) as follows: 1/3 volume resin and 2/3 volume acetone for 15 min, 1/2 volume resin and 1/2 volume acetone for 30 min, 2/3 volume resin and 1/3 volume acetone for 3 h, and pure resin overnight. Subsequently, samples were transferred in resin-filled tubes and polymerized in an oven at 65 °C for two days.

Ultrathin sections (90 nm) of the grains were cut by using an ultramicrotome LEICA EM UC7 (Wetzlar, Germany) and a diamond knife type “ultra 45°” (DIATOME Ltd., Switzerland), collected on copper grids (300 mesh) and stained with 2.5% gadolinium triacetate for 30 min and 3% lead citrate for 8 min. Grids were analyzed at 120 kV with a TEM Zeiss Libra 120 equipped with a LaB_6_ (Lanthanum-Hexaboride) cathode. Images were acquired using a side port camera Morada G2, 11 MP (Soft Imaging System GmbH, Münster, Germany) and a bottom mount camera Sharp:eye TRS (2 × 2 KP) with iTEM software and processed using Adobe Photoshop CS5.

## Results

### Multivariate statistics unravel proteome reorganization across multiple biological processes during barley grain development

**Figure 1:**
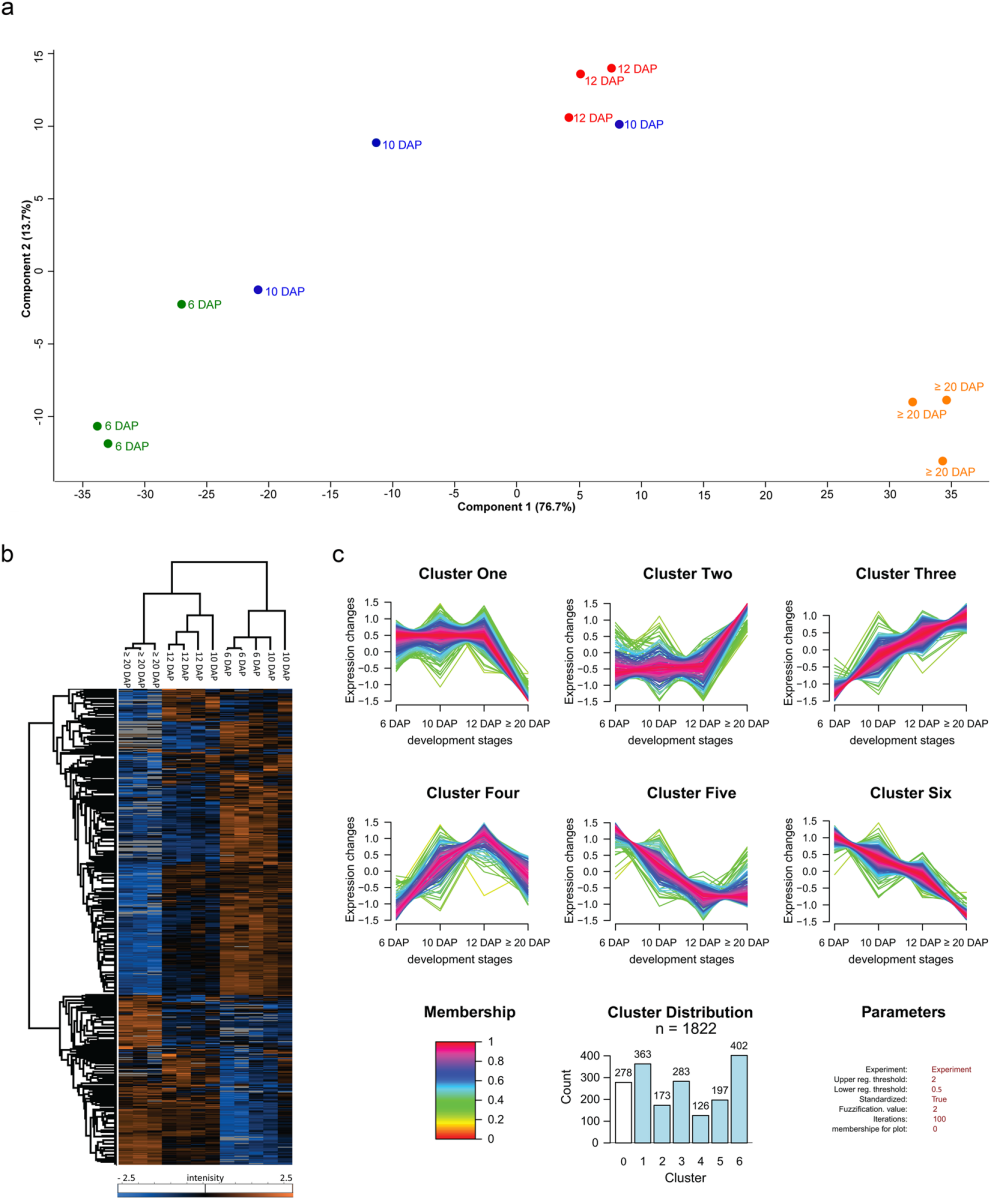
Proteome profiling during barley grain development. (**a**) PCA was conducted on logarithmically transformed protein intensities; each dot corresponds to a single biological replicate (n = 3). (**b**) Hierarchical cluster analysis of quantified proteins along barley grain development was performed with Perseus after Z-score transformation of the data^21^. Clustering of proteins was done based on Euclidian distance while samples’ clustering is based on Pearson correlation. (**c**) Cluster of proteins dynamics along the grain development. Quantified proteins were subjected to unsupervised clustering with the fuzzy c-means algorithm implemented in GproX^24^. Cluster distribution indicates the number of proteins in each cluster. Membership value represents how well the protein profile fits the average cluster profile.

To identify proteins that are localized at the endomembrane system and/or are functionally associated with the rearrangement of the endomembrane system in barley, grains of different development stages including 6, 10, 12 and ≥20 DAP were harvested as previously described^16^. We used liquid chromatography-mass spectrometry (LC-MS/MS) to identify a total of 3,005 proteins. Our label-free quantification (LFQ) data obtained by MS was validated: first, by the comparison of the relative intensity of the HveF1a and HvVSR1 to a semi-quantitative Western blot (Supplementary Fig. S2b, Supplementary Table S2) that both show the stable and increasing trend of protein accumulation, respectively. Second, the measured protein abundances were highly reproducible with an average Pearson’s correlation coefficients of >0.95 between biological replicates (Supplementary Fig. S2c). Additionally, the performed Principal Component Analysis (PCA) supports these results and shows that samples grouped and clustered in a stage-specific manner (Fig. 1a). However, two samples of 10 DAP cluster together with samples of 6 and 12 DAP, respectively. The high distribution of the standard deviation of the normalized LFQ intensities at 10 DAP (Supplementary Fig. S2d) point to a physical variability at 10 DAP. Additionally, the seed weight between the stages are significantly different except the stage between 6 and 10 DAP (Supplementary Fig. S2e) These results are in line with the previously published microscopic data where the most dramatic endomembrane rearrangements could be observed between 8 and 12 DAP^13^.

A total of 1,822 out of 3,005 were quantified in at least 9 out of the 12 analyzed biological samples (Supplementary Table S2). To determine the proteins that were significantly changed along the grain development, we applied a one-way ANOVA analysis, corrected with a permutation-based false discovery rate (FDR) (*p* < 0.05). Among the 1,822 quantified proteins, the abundance of 1,544 proteins was significantly changed (Supplementary Table S2). To assess the proteome dynamics during barley grain development, both PCA and a hierarchical bi-clustering analysis (HCA) were performed. Thereby identical clusters of stage-specific groups related to development stages of the barley grain were identified (Fig. 1a,b). An investigation of the PCA results shows that PC1 (76.7% of variance) separated the sample based on the different development stages, particularly between the early (6 DAP) and late development stages (≥20 DAP). PC2 (13.7% of variance) was rather more involved in the early development stage discrimination. Protein loadings on each principal component are indicated in Supplementary Table S2. As expected, SSPs were among the highest loadings on PC1. Interestingly, most of the detected changes occurred at ≥20 DAP (Fig. 1b).

To refine our protein expression pattern analysis, unsupervised clustering was performed with GproX software to partition the temporal profiles of 1,544 significantly changed proteins measured at all time points. Six clusters based on a fuzzy-mean clustering process^24^ were detected (Fig. 1c and Supplementary Table S2):

Proteins presenting a higher expression level at 6 than at ≥20 DAP belong to Clusters One, Five and Six. Those three clusters account for most of the significantly changed proteins (in total, 962 proteins). It is known that endosperm development involves cell division, cellular differentiation events and the deposition of SSPs between early, late and mid-development^30,31^. These findings were confirmed by toluidine-stained sections prepared at 6, 12 and ≥20 DAP that revealed fully cellularized endosperm including three aleurone cell layers at 6 DAP (Supplementary Fig. S3a). Cluster Four accounts for 126 proteins and shows its highest protein abundance between 10 and 12 DAP, which corresponds to mid-stage, where the differentiation of aleurone, subaleurone, and starchy endosperm is finalized (Fig. 1c and Supplementary Fig. S3a). Finally, the 456 proteins associated with Cluster Two and Three exhibit higher expression levels at ≥20 than at 6 DAP, corresponding to the end of mid-stage and beginning of the late stage, where the accumulation of storage reserves can be observed in the endosperm (Fig. 1c and Supplementary Fig. S3). Using LC-MS/MS, all 20 identified SSPs showed significantly higher abundance at ≥20 DAP (Supplementary Fig. S3b,c).

Toluidine staining of sections prepared at 6, 12 and ≥20 DAP confirmed protein accumulation starting at 12 DAP and increasing at ≥20 DAP in subaleurone as well as in the starchy endosperm (Supplementary Fig. S3a). Additionally, Cluster Two and Three contain HvPDIL1–1 and HINs^15,16^.

### Development phase I contains proteins of high abundance associated with endocytosis and cytoskeleton, plasma membrane proteins, and ATPases

Based on a BLAST search, 94 proteins related to the endomembrane system (out of the 1 544 significantly changed ones) could be identified in total, where 59 compartment-specific proteins (secretory pathway, peroxisome, plasma membrane, sorting, transport, degradation, and vacuolar processing) and 35 trafficking regulators were defined (dynamins, SNAREs, disulfide-generating enzyme and- carrier, ATPase, GTPase, GTPase-activating protein, RAB regulator, and GTP binding protein) (Table 1). The abundance of all identified endomembrane related proteins at 6, 10, 12, and ≥20 DAP were visualized by a heat map categorizing the protein expression pattern based on Pearson correlation (Supplementary Fig. S4). Compartment-specific proteins and trafficking regulators were categorized in pink and blue, respectively (Supplementary Fig. S4).

The 94 proteins could be associated with the three main development phases (Supplementary Fig. S4): 39 proteins present a higher expression during the development phase I (green cluster), 15 proteins presented an expression peak at the development phase II (yellow cluster), and 40 proteins are associated to the development phase III (red cluster). Within each phase, compartment-specific proteins and trafficking regulators were categorized (Table 1, Supplementary Fig. S5a,b). Taken together, these data present molecular regulators for the endomembrane system in developing barley grain.

**Figure 2:**
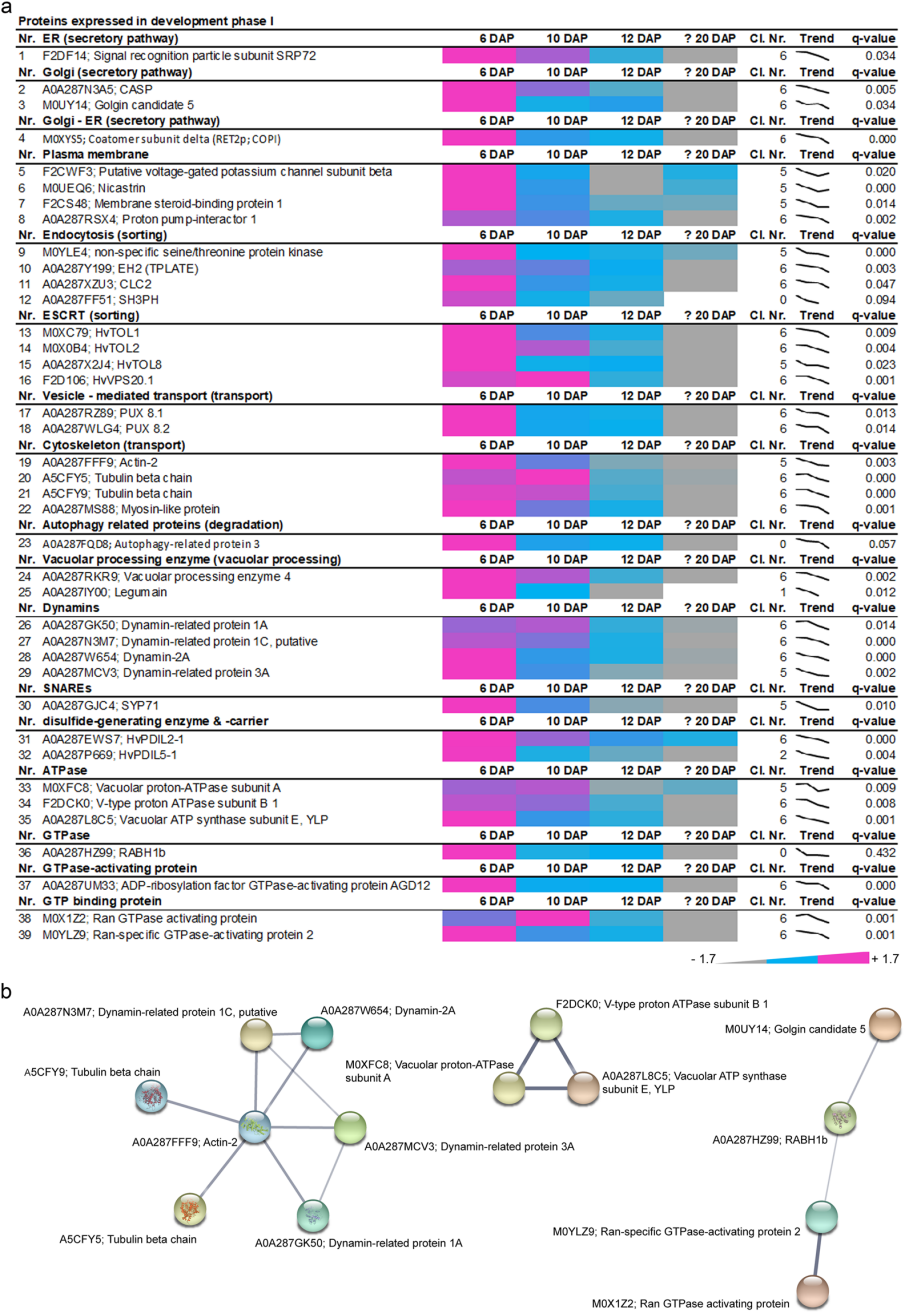
Identification of proteins that are highly abundant at development phase I of developing barley grains. (**a**) Data-matrix heat map representing Z-score values of 6, 10, 12 and ≥20 DAP. Heat map was prepared using Microsoft Excel. Scale: grey = smallest value; blue = 50% quantile; pink = highest value. (**b**) Proteins present in this stage were analyzed using the STRING database. STRING default parameters were used^25^, protein names are indicated. PBs are identified in the bright field as small spherical structures as recently published^13,15,16^.

Among the 39 proteins identified in the development phase I, proteins related to the secretory pathway (ER, Golgi, Golgi – ER), plasma membrane, sorting pathway (endocytosis, ESCRT), transport (vesicle-mediated transport, cytoskeleton), degradation (Autophagy-related protein 3) and to vacuolar processing as well as trafficking factors (dynamins, SNAREs, disulfide-generating enzymes and carriers, ATPase, GTPase, GTPase-activating protein and GTP binding protein) were enriched at 6 and 10 DAP (Fig. 2a).

**Figure 3:**
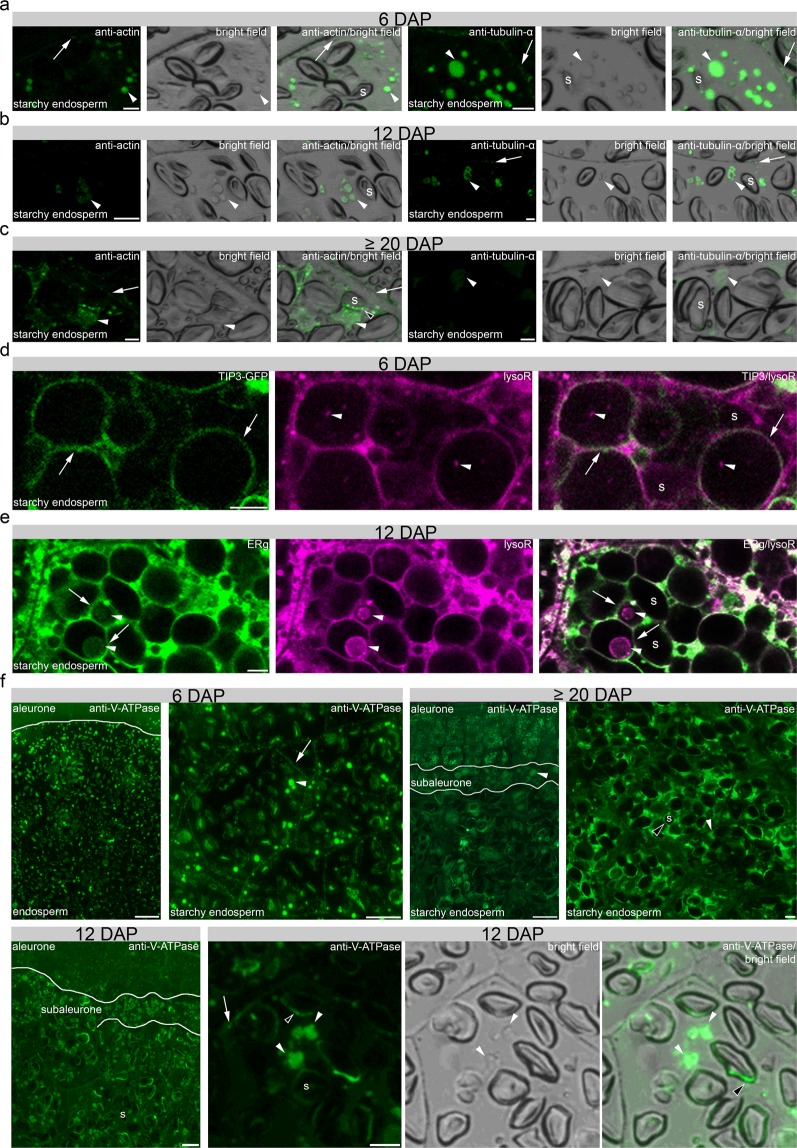
*In situ* microscopical analyses of the cytoskeleton and acidification of PBs in development phase I. (**a–c**) Immunofluorescence studies of 1.5 µm prepared sections of 6, 12 and ≥20 DAP using antibodies for anti-actin and anti-tubulin-α showing a strong signal at PBs (arrowheads), respectively. PBs are identified in the bright field as small spherical structures a recently published^13,15,16^. Note the signal at the plasma membrane with anti-tubulin-α (arrow). The fluorescence signal intensity is weaker at 12 and at ≥20 DAP. Note the additional signal at the periphery of the starch granule at ≥20 DAP using anti-actin (black-white arrowhead). (**d**) LysoTracker Red (lysoR) accumulation (arrowheads) within TIP3-GFP labelled vacuoles (arrows) at 6 DAP. (**e**) ER-Tracker Green (ERg)-labelled compartments (arrows) accumulate LysoTracker Red (lysoR) positive PBs (arrowheads) at 12 DAP. (**f**) Immunofluorescence studies of 1.5 µm sections of 6, 12 and ≥20 DAP using anti-V-ATPase antibody showing no positive signal at aleurone at 6 DAP whereas strong signal could be detected in aleurone at ≥20 DAP. In starchy endosperm, anti-V-ATPase antibody labels strongly PBs (arrowheads) and was found weaker at the plasma membrane (arrows). At 12 DAP, signal appeared at PBs in subaleurone and starchy endosperm. Note the specific signal at the PBs (arrowheads), at the periphery of starch granules (black-white arrowhead) and the weak labelling of vesicles at the plasma membrane (arrow). At ≥20 DAP, the anti-V-ATPase antibody labels strongly PBs in subaleurone (arrowhead), but to lesser extent in the starchy endosperm. s = starch granule. Bars = 5 µm in **a–e** and 10 µm in **f**, except at ≥20 DAP where the bar represents 100 µm in the overview picture.

More precisely, plasma membrane-associated proteins such as putative voltage-gated potassium channel subunit beta (F2CWF3), Membrane steroid-binding protein 1 (F2CS48), Nicastrin (M0UEQ. 6) and Proton pump-interactor 1 (A0A287RSX4) present decreasing abundance in concomitance to proteins associated to the endocytosis processes such as dynamins (A0A287GK50, A0A287N3M7, A0A287W654, A0A287MCV3), tubulin (A5CFY5, A5CFY9), actin (A0A287FFF9), myosin (A0A287MS88), EH2 (A0A287Y199) and CLC2 (A0A287XZU3). In line with the constant need of energy necessary for endocytosis and plasma membrane remodeling processes, three vacuolar-ATPase subunits were identified (A: M0XFC8, B1: F2DCK0, E: A0A287L8C5). All three subunits showed a decreased expression, indicating an acidification process at the early development stage (Supplementary Table S2). Using STRING, a functional association between dynamins and cytoskeleton-related proteins was visualized (Fig. 2b), both necessary for plant endocytic processes^32,33^. Additionally, STRING revealed a functional correlation between several ATPases that were abundant at 6 and 10 DAP (Fig. 3b). The functional association between Golgin candidate 5, RABH1b and further GTPases shown by STRING points to an active protein sorting in the trans-Golgi or at the trans-Golgi network (TGN)^34^. Taken together, the proteins that are highly abundant at 6 DAP point to the necessity of cytoskeleton, acidification, and sorting during the development phase I.

We performed immunofluorescence studies of actin and tubulin to follow the subcellular appearance of the cytoskeleton during barley endosperm development. We observed strong signals within PBs at 6 DAP, becoming weaker at 12 and ≥20 DAP, respectively (Fig. 3a–c). PBs are identified in the bright field as small spherical structures as recently published^13,15,16^. Interestingly, a faint actin signal was observed at the plasma membrane at ≥20 DAP, whereas the signal of tubulin at the plasma membrane was strong at 6 DAP but was reduced at 12 and ≥20 DAP (Fig. 3a–c). No signal could be detected in the negative controls for immunofluorescence for all stages (Supplementary Fig. 6b).

Recently it was shown that PBs in maize are acidified and can be visualized by live cell imaging of fluorescent organelle markers^35^. To determine if the identified and functionally associated ATPases are putatively involved in the acidification of PBs in barley, we first used LysoTracker Red to detect acidic compartments in the transgenic TIP3-GFP line that visualizes PSVs. At 6 DAP, acidic compartments could be observed within PSVs in the starchy endosperm (Fig. 3d). Large vacuoles are most prominent in the starchy endosperm at 6 DAP^13^ when the accumulation of PBs has just started (Supplementary Fig. 3)^15^, indicating that the LysoTracker Red-labelled compartments represent PBs. Indeed, co-labelling of ER and acidic compartments in starchy endosperm cells at 12 DAP revealed LysoTracker Red- and ER-Tracker Green-positive PBs (Fig. 3e).

To determine the time-dependent subcellular distribution of ATPase, we used immunofluorescence microscopy with anti-V-ATPase subunit epsilon antibody on sections at 6 and ≥20 DAP (Fig. 3f). V-ATPases are described to localize at the membranes of PSVs as well as in mature PB in developing pea cotyledons^36^. Whereas at 6 DAP a punctate structure was observed at the plasma membrane and a strong signal within PBs, a weaker labelling could be observed at the periphery of starch granules and within PBs at 12 DAP (Fig. 3f). At ≥20 DAP, an additional strong signal appeared in aleurone (putatively at the tonoplast of PSVs), whereas the signal was weak within PBs and at the periphery of starch granules in the starchy endosperm (Fig. 3f). No signal could be detected in the negative controls for immunofluorescence for 6, 10, 12 and ≥20 DAP (Supplementary Fig. 6b). Overall, our proteomics and *in situ* microscopic results point to a high abundance of proteins involved in cytoskeleton regulation and acidification of PBs at early barley grain development stage.

### Sorting-associated proteins preferentially accumulate at development phase II

**Figure 4:**
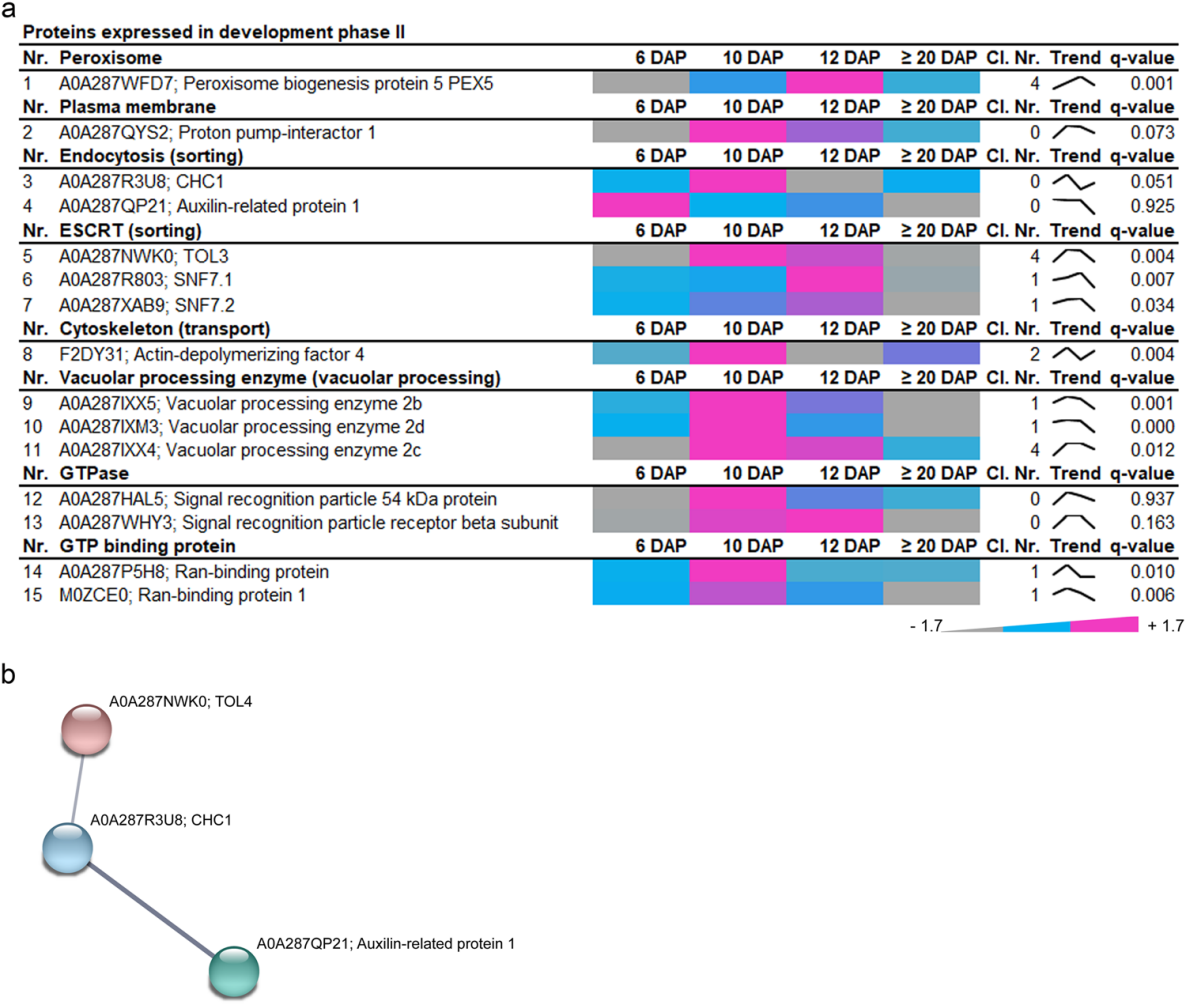
Identification of highly abundant proteins at development phase II of developing barley grains. (**a**) Data-matrix heat map representing Z-score values of 6, 10, 12 and ≥20 DAP. Heat map was prepared using Microsoft Excel. Scale: gray = smallest value; blue = 50% quantile; pink = highest value. (**b**) Proteins present in this stage were analyzed by STRING database. STRING default parameters were used^25^, protein names are indicated.

Among the 15 proteins associated with development stage II (Fig. 4a, Supplementary Fig. S4, yellow tree), we found one peroxisome protein, PEX5 (A0A287WFD7); one cytoskeleton protein, Actin-depolymerizing factor 4 (F2DY31); proteins related to endocytosis, CHC1 (A0A287R3U8), Auxilin-related protein 1 (A0A287QP21); as well as proteins of ESCRT machinery, vacuolar processing enzymes (VPEs), GTP-binding proteins, GTPases, and ATPases (Fig. 4a). Again, three ESCRT proteins, TOL4 (A0A287NWK0), SNF7.1 (A0A287R803) and SNF7.2 (A0A287XAB9) were identified, pointing to sorting processes including MVBs already detected in development phase I.

Using STRING, a functional association between the ESCRT TOL4, clathrin, and an auxilin-related protein appeared (Fig. 4b). TOLs have putative clathrin-binding motifs^37^ and auxilin-related protein 1 functions as clathrin-uncoating factor^38^, and both were described to be involved in endocytosis. Interestingly, proteins involved in sorting, transport, and degradation have been already identified in the development phase I.

### Compartment-specific proteins and trafficking regulators participating in storage protein targeting, transport, and deposition are accumulating at development phase III

**Figure 5:**
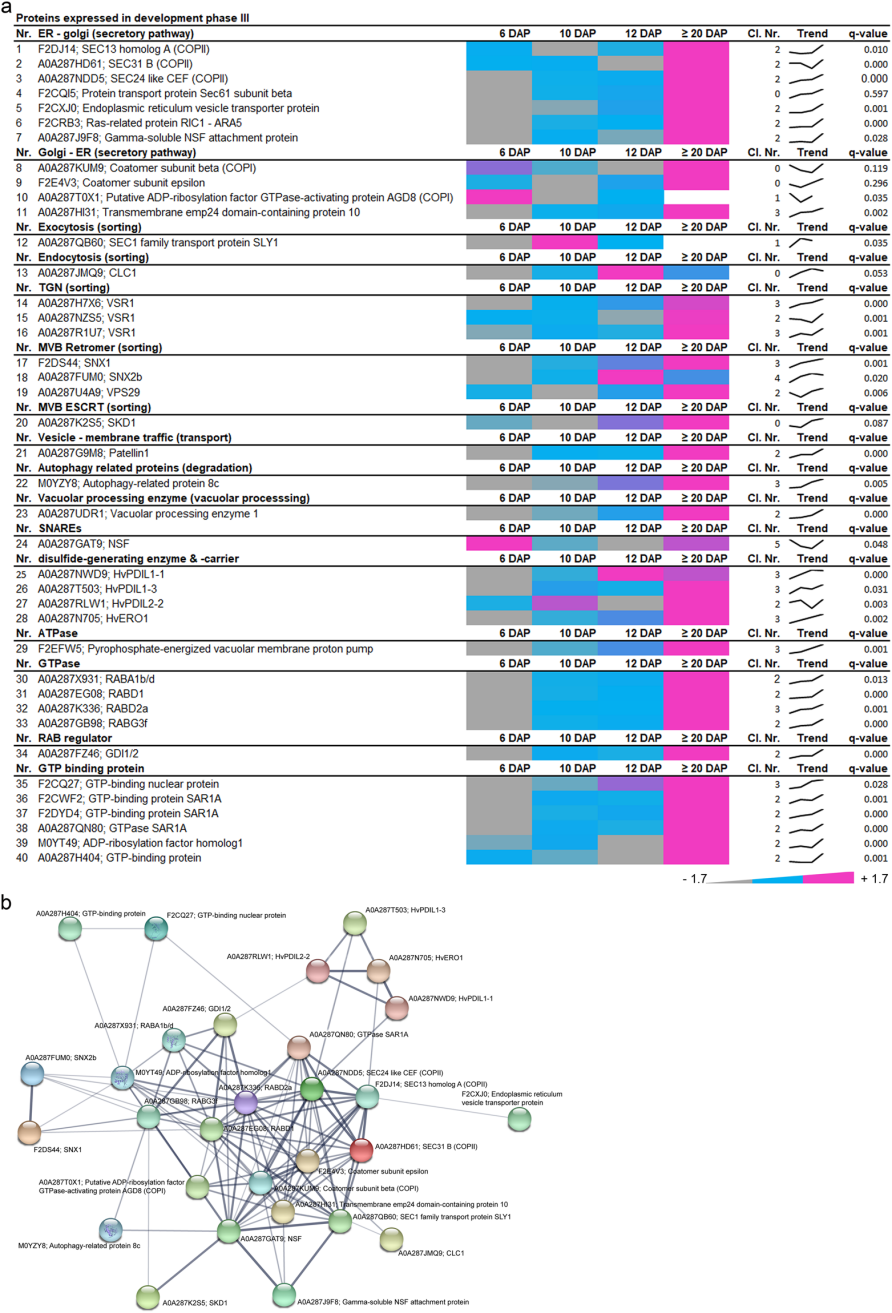
Identification of highly abundant proteins at development phase III of developing barley grains. (**a**) Data-matrix heat map representing Z-score values of 6, 10, 12 and ≥20 DAP. Heat map was prepared using Microsoft Excel. Scale: gray = smallest value; blue = 50% quantile; pink = highest value. (**b**) Proteins present in this stage were analyzed by STRING database. STRING default parameters were used^25^, protein names are indicated.

In total 40 proteins were associated with development phase III, most of them highly abundant at ≥20 DAP (Fig. 5a). Interestingly, 11 proteins involved in the secretory pathway (e.g., COPII/COPI), and 9 proteins that function in the protein sorting (e.g., VSR1, retromer, and ESCRT) were identified. Additionally, proteins involved in degradation (autophagy-related 8c, vacuolar processing enzyme1) were highly abundant at ≥20 DAP. Among other proteins, one SNARE was identified as well as 11 trafficking regulators associated with the ATPase, GTPase or the GTP-binding group. Strikingly, endomembrane-associated proteins characterizing the phase III presented the highest interconnectivity within the STRING database compared to phases I and II (Fig. 5b).

As expected, important ER markers such as the disulfide isomerase protein (HvPDIL1-1 and ERO1) were found in this group, being in line with the accumulation of SSPs (Supplementary Fig. S2). The STRING network indicates that the group of ER markers is linked with proteins associated with the secretory pathway and their associated factors such as a RAB regulator GDI1/2 (A0A287FZ46), COPII (F2DJ14, A0A287HD61, A0A287NDD5) as well as COPI proteins (A0A287KUM9, A0A287T0X1) and other regulators (F2E4V3, A0A287HI31). Interestingly, this core set of proteins are functionally associated with diverse proteins including ESCRT related SKD1 protein (A0A287K2S5), autophagy-related protein 8c (M0YZY8), sorting nexins, as well as regulatory factors related proteins (GTPases, GTP binding proteins).

However, some of the identified functional groups clustered within each development phase of barley grain development, suggesting a temporal regulation of the identified mechanisms. For example, VSR1, which participates in vacuolar sorting of 12S globulins and 2S albumins in *A. thaliana* seeds^39^, was strongly localized in the aleurone layer instead of the starchy endosperm at ≥20 DAP (Supplementary Fig. 7a), indicating predominantly functional activity in the aleurone layer during development or during germination. The vacuolar processing enzymes (VPEs) are known to be involved in PCD^40–42^ as well as in processing SSPs in seeds^43–45^. Similarly, ESCRT-related proteins contained members of each development phase, indicating tissue-specific functions of proteins.

### ESCRT-III HvSNF7 associates to MVBs at development phase I and both localize to and within PBs at development phase II and III

**Figure 6:**
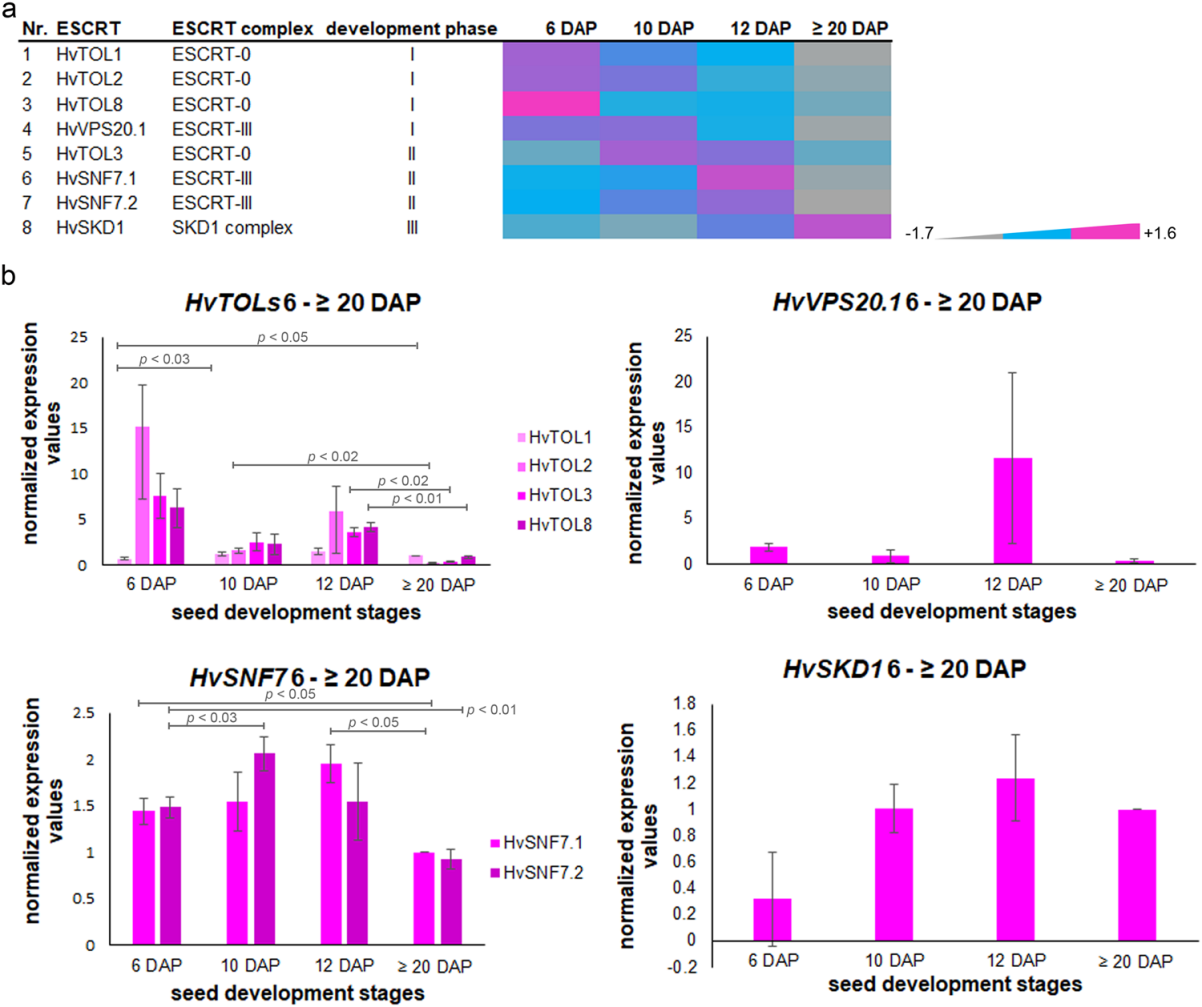
Temporal regulation of identified ESCRT proteins and transcripts. (**a**) Data-matrix heat map representing Z-score values of 6, 10, 12 and ≥20 DAP. Heat map was prepared using Microsoft Excel. Scale: grey = smallest value; blue = 50% quantile; pink = highest value. HvTOL1, HvTOL2, HvTOL8, and HvVPS20.1 show a high abundance at developmental phase I, but a continuous decrease over the development of barley grain. HvTOL3, HvSNF7.1, and HvSNF7.2 exhibit an expression peak during developmental phase II, while HvVPS4 continuously accumulated during the early grain development up to developmental phase III. (**b**) Temporal quantification of *HvESCRT* transcripts in developing barley grains. Bar graphs describe the average over three biological replicates of the normalized transcripts from *HvTOL1/HvTOL2/HvTOL3/HvTOL8*, *HvVPS20.1*, *HvSNF7.1*/*HvSNF7.2* and *HvSKD1* at 6, 10, 12 and ≥20 DAP. For statistical analyses we performed a Student’s t-test (n = 3). Bars represent standard deviation; *p-*values are indicated.

Proteomic analysis identified eight proteins related to ESCRT-0, ESCRT-III and SKD1 complex. Interestingly, they showed different expression patterns and subsequently belonged to different clusters (Fig. 6a).

To gain insight into theexpression behavior of identified ESCRT members, we analyzed the transcript levels of *HvTOLs*, *HvSNF7s*, *HvVPS20.1*, and *HvVPS4* during barley grain development by RT-qPCR (Fig. 6b). RNA was isolated from whole grains harvested at 6, 10, 12 and ≥20 DAP, and the previously characterized most stable genes were used to normalize the *ESCRT* transcripts^16^. A high correlation between transcript and protein abundances for all identified HvESCRT members was observed, except for *HvVPS4*. Even though the Hv*VPS4* transcript follows the same trend as *HvSNF7* transcripts and proteins, HvVPS4 protein increased, suggesting a delay of the response or a fine-tuning of HvVPS4 translation (Fig. 6b). These results indicate that the expression of HvESCRT is temporally regulated during barley grain development.

**Figure 7:**
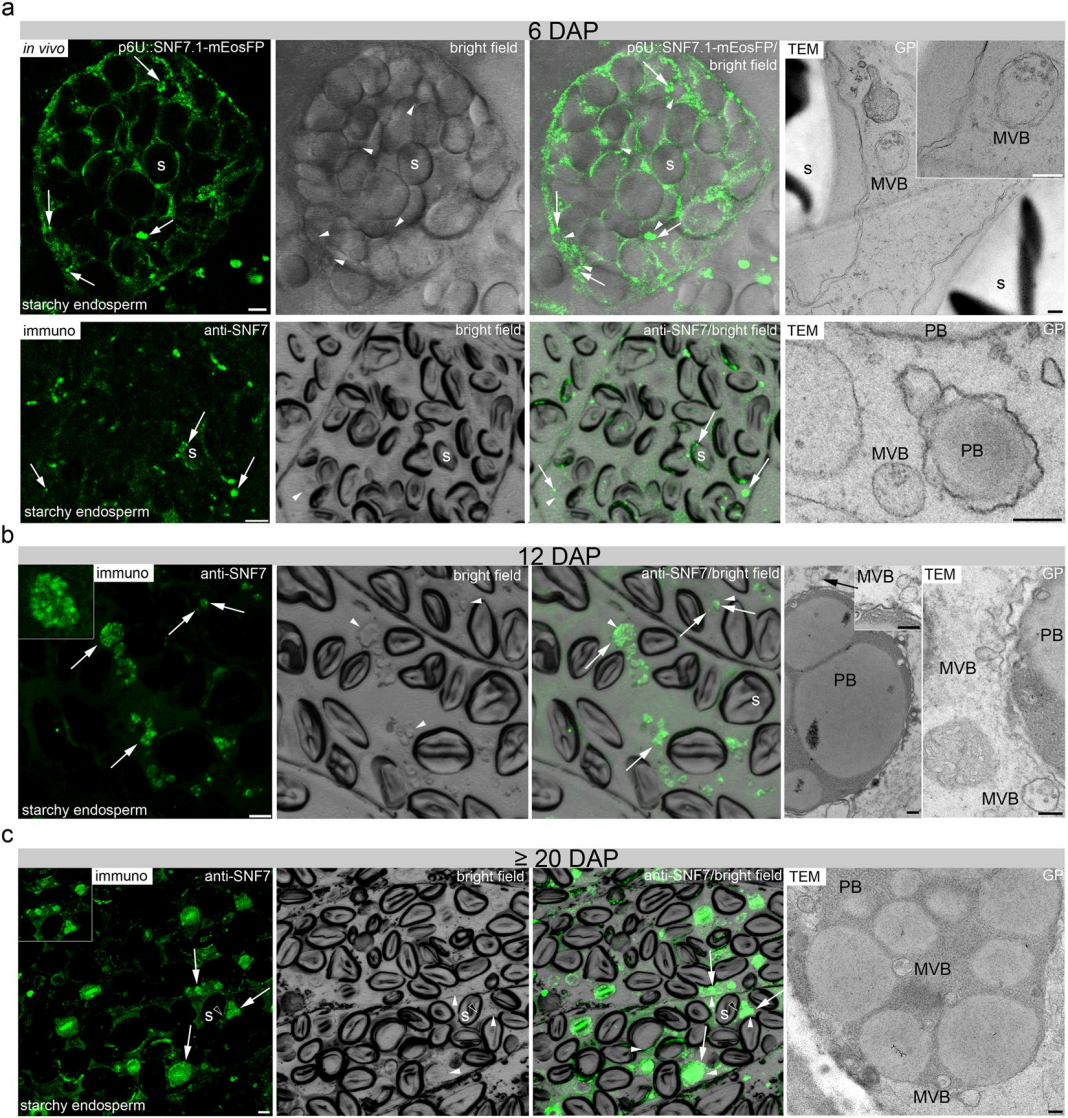
Localization of HvSNF7 and MVBs in developing barley grains. (**a**) Confocal live cell imaging of p6U::SNF7.1-mEosFP and immunofluorescence study using anti-SNF7 at 6 DAP showing both punctate structures (arrows) around protein PBs (arrowheads) and starch granules (indicated by the index s). Note the MVB between two starch granules in the TEM section (ca. 90 nm in thickness) and nearby PBs at 6 DAP. (**b, c**) Immunofluorescence studies of 1.5 µm sections of 12 and ≥20 DAP using anti-SNF7 showed weak/strong punctate structures (arrows) at PBs/within PBs (arrowheads) at 12 DAP whereas a more diffuse signal (arrows) within the PBs (arrowheads) could be detected at ≥20 DAP. At 12 DAP, a MVB approximates to a PB (left and right image) and fuses with a membrane surrounding a PB (right image). MVBs are detected within fused PBs at ≥20 DAP. Scale = 5 µm, except in TEM images = 250 nm. PBs are identified in the bright field as small spherical structures as recently published^13,15,16^.

ESCRT originally refers to a protein–protein interaction network in yeast and metazoan cells that coordinates sorting of ubiquitinated membrane proteins into intraluminal vesicles (ILVs) of the MVB^46–48^. Specifically, ESCRT-III is known to be necessary for membrane remodeling that drives the biogenesis of MVBs^49,50^. Recent electron tomography studies in *A. thaliana* revealed that intraluminal vesicles form as large networks of interconnected or concatenated vesicles. AtSNF7 was detected in the intervesicle bridges, suggesting that ESCRT-III proteins remain trapped inside the vesicle cluster in MVBs and are finally delivered together with the cargo into the vacuole^51–53^. So far, no observations of HvSNF7 and MVBs have been reported in barley endosperm tissues. Thus, the subcellular localization of HvSNF7 and the identification of MVBs was performed. To elucidate the subcellular localization of HvSNF7, we first studied the localization of HvSNF7 *in vivo* at the early development stage using the transgenic line p6U::SNF7.1-mEosFP. Confocal live cell imaging of the p6U::SNF7.1-mEosFP transgenic line revealed punctual structures and few agglomerations around PBs at 6 DAP (Fig. 7a). AtSNF7.1 is known to form homodimers and thus can possibly lead to agglomerations^54^. Indeed, bimolecular fluorescence complementation (BiFC) and Yeast Two-Hybrid (Y2H) analyses revealed homodimerization of HvSNF7.1 (Supplementary Fig. 8a,b). We used an anti-SNF7 antibody to analyze the localization by immunofluorescence microscopy at 6 DAP where the observations of small punctual structures and few agglomerations from the live cell imaging could be confirmed (Fig. 7a). Transmission electron microscopy (TEM) analyses showed that grains at 6 DAP contained MVB’s in proximity to starch granules and PBs, as representatively displayed in Fig. 7a. As live cell imaging in developing endosperm is limited to early- and mid-development stages^13^, immunofluorescence analyses of sections prepared from 12 and ≥20 DAP were analyzed. Strong signals within PBs at 12 and ≥20 DAP could be detected (Fig. 7b). The punctured structures that could be observed inside the PBs possibly appeared by fusing of several smaller PBs (Fig. 7b). Additionally, a weak signal could be observed at the periphery of starch granules at 12 and ≥20 DAP (Fig. 7b). Indeed, TEM analyses identified MVBs associated with PBs at 12 DAP and within fused PBs at ≥20 DAP (Fig. 7b). These findings indicate that HvSNF7 is first localized at vesicular structures (putatively at MVBs) and later associated with single PBs and finally localized inside fused PBs.

## Discussion

### Proteomics and *in situ* microscopic analyses enable the mapping of the endomembrane system of developing barley endosperm

Many proteomic analyses in barley grain allow us to identify and understand the molecular composition of developing barley grain^55–57^. However, less is known about proteins regulating the endomembrane system in developing barley grains. Only one of the proteins identified in our study, Membrane steroid-binding protein 1 (F2CS48), was recently characterized in barley^58^. Recent studies have shown that the endomembrane system and SSP trafficking are spatio-temporally regulated in developing barley endosperm between 8 and 12 DAP^13,15^. This time-dependent regulation was confirmed by PCA analysis of our proteomics data, which revealed stage-specificity of protein expression during grain development.

**Figure 8:**
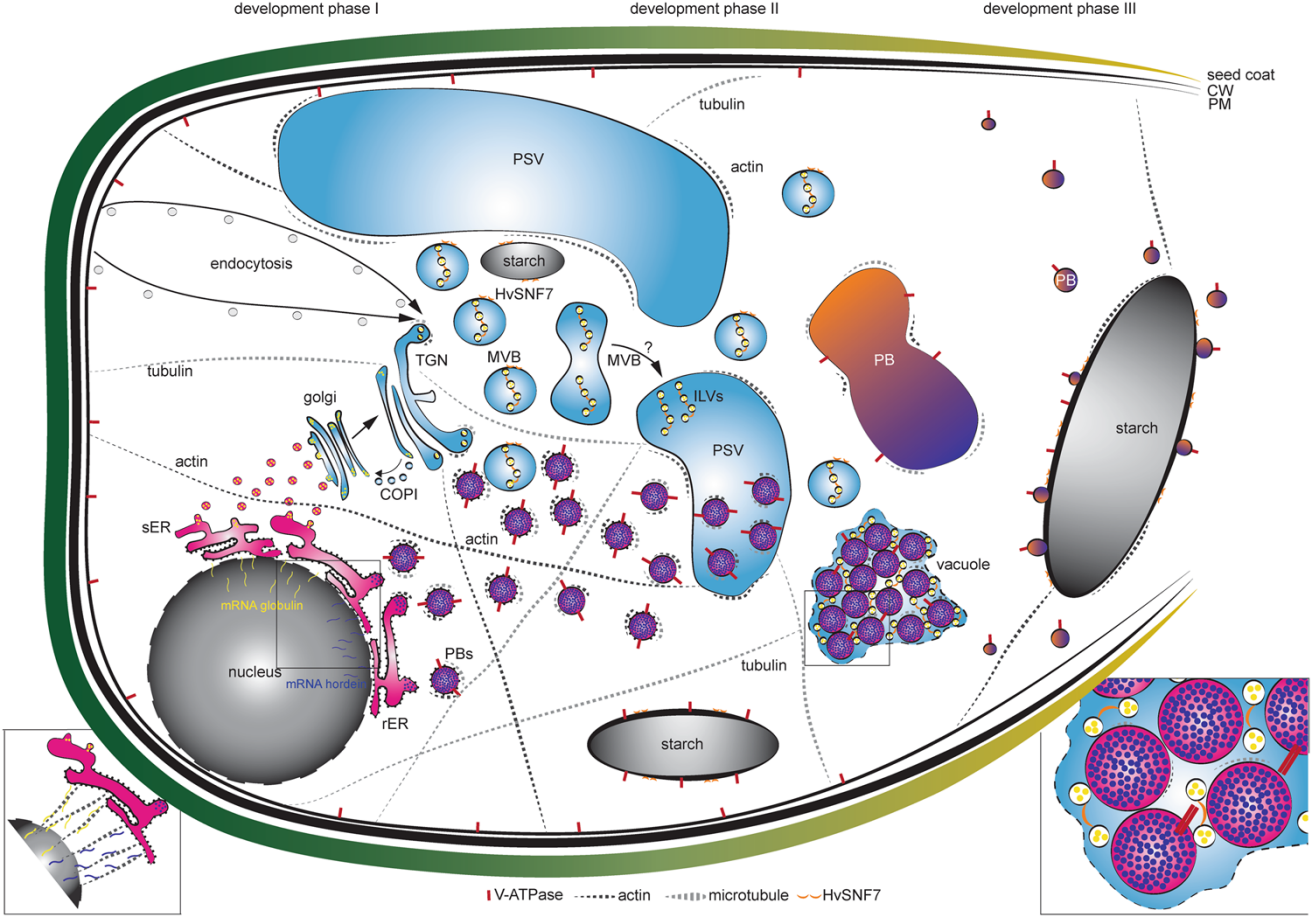
Quantitative *in situ* mapping of the endomembrane system during barley endosperm development. Quantitative proteomics and *in situ* microscopic analyses identified HvSNF7 and MVBs as putative key players for protein sorting into PBs during barley starchy endosperm development. At developmental phase I, mRNA of e.g., globulin and e.g., hordein are transported by the cytoskeleton to the ER where they are entering different protein trafficking pathways (zoom in)^59–63^. During developmental phase I and II, PSVs become smaller and PBs are formed, both putatively regulated by the cytoskeleton. In parallel, MVBs containing HvSNF7 appear and possibly fuse with PSVs, leading to PSVs containing HvSNF7 positive ILVs and PBs (zoom in). Between developmental phase II and III, PSVs collapse, and PBs fuse to one big PB containing HvSNF7. At developmental phase III, PBs become smaller again, attaching to the protein matrix at the periphery of the starch granule. Note the additional localization of HvSNF7 at the starch granules between phase I and III. Additionally, V-ATPase localize to PBs at developmental phase I, acidifying PBs. V-ATPase could be further observed at starch granules during development. Schema is not in scale. PSV, protein storage vacuole; MVB, multivesicular body; ILVs, intraluminal vesicles; PB, protein body; sER, smooth ER; rER, rough ER; CW, cell wall; PM, plasma membrane.

A detailed analysis of the 94 endomembrane related proteins associated them with three development phases: I, II and III. Altogether with previously published work, correlation of proteomics data with *in situ* microscopic analyses allowed us to provide the first temporal map of endomembrane-related proteins involved in stage-specific regulation of different endomembrane processes (Fig. 8): in development phase I, prolamin and glutelin RNAs are localized to two subdomains of the cortical ER, and targeted to the Golgi or to PBs by RNA-binding proteins and the cytoskeleton^59–63^. Additionally, development phase I displays endocytic activities involving plasma membrane rearrangement. Such processes have been shown to be associated with specific cytoskeleton dynamics: besides the necessity of the plant cytoskeleton in vesicle trafficking and organelle movement^64^, actin is required for the auxin-dependent convolution and deconvolution of the vacuole^65^.

Specifically, SNAREs, actin and its associated motor protein myosin shape the vacuole by actin-dependent constrictions. While the protein accumulation starts at 6 DAP with observable PBs at 8 DAP, the size of PSVs decreases between 8 and 10 DAP. As we could observe actin as well as tubulin associated with PBs, we conclude that the cytoskeleton proteins present at early stages of barley endosperm development regulate the PBs formation/trafficking and the size of PSVs. Concurrent with the endocytic activity, microscopic analyses indicate an increase of the acidity within PBs. During development phase II, processes associated with the sorting system seem to be highly active. Recently, MVBs were discussed to be taken up into the vacuole by autophagy^66^. Additionally, studies in *A. thaliana* root cells have shown that central vacuoles were derived from MVB to small vacuole transition and subsequent fusions of small vacuoles^67^.

Thus, we propose that MVBs loaded with HvSNF7 possibly contribute to PSV rearrangement events, resulting in large PSVs containing PBs associated with MVBs and HvSNF7, as it is known that PBs are taken up by PSVs at 10 DAP^13^. Finally, during development phase III, PBs, MVBs, and HvSNF7 were found in proximity to the protein matrix at the periphery of starch granules, while the presence of the cytoskeleton was reduced.

Here, we point out the necessity to correlate proteomics data with microscopic analysis for reasons of spatio-temporal specificity. Although the bulk of the barley grain is mainly occupied by the starchy endosperm, we cannot exclude that identified proteins can reflect different spatial activity. For example, VPE4 was described to be most expressed in the pericarp of barley between 8 and 10 DAP^68^ and to be necessary for programmed cell death execution in the developing pericarp^42^, thereby being responsible for the grain size, starch and lipid content. As our proteomics data identified VPE4 to be most abundant at 6 and 10 DAP and subsequently to be grouped into development phase I, we assume the main function of VPE4 is in the pericarp. In contrast, VPE2b, c, and d, which are most abundant between 10 and 12 DAP and were grouped to development phase II, have been described to be involved in nucellar PCD^68^. Thus, proteomics from dissected sections would be useful to identify tissue-specific proteins. In order to refine our analysis to a spatio-temporal level, we compared our dataset with previously published LMD-based proteomics analysis^15^. However, only four out of our 94 identified endomembrane related proteins could be detected (F2DY31, Actin-depolymerization factor 4; A0A287NWD9, HvPDIL1-1; A0A287RLW1, HvPDIL2-2; and F2CQ27, GTP-binding nuclear protein). This indicates a detection range limitation of proteomic analyses of dissected samples prepared for laser microdissection. Consequently, a combined analysis of proteomics together with microscopy appears as the most appropriate strategy.

It is worth mentioning that our data provide comprehensive coverage of the endomembrane-associated proteins involved in the rearrangement of the endomembrane system and protein trafficking. However, we cannot entirely exclude additional proteins involved in these processes or organelle-specific proteins involved in other processes. For example, even though the importance of the Golgi in protein trafficking was shown previously in wheat in the transition between stage I and II^69–71^, only two Golgi-associated proteins could be found (Golgin 5 and CASP), both grouped to development phase I.

In order to define if Golgi-associated proteins are underrepresented in our dataset, we compared our data with previously identified proteins in *A. thaliana*^72^. Interestingly, we could explicitly identify more than 10 Golgi-resident proteins such as cell wall synthesis-associated proteins (e.g., UDP-glucuronic acid decarboxylase, A0A287H8Z0), which are parts of the glycosylation processes (Supplemental Table 2). It is possible that these identified proteins are active in aleurone, as previous data characterized the barley aleurone *N*-glycoproteome, in which numerous *N*-glycosylation sites were identified that play key roles in protein processing and secretion^73^.

### How do ESCRTs and MVBs contribute to PB formation

Although MVBs were reported to be responsible for targeting proteins to the storage vacuole in maize aleurone cells^74,75^, their existence and localization during cereal endosperm development has so far not been investigated. Here, our proteomics data revealed VPS29, SNX1, and SNX2b, proteins from the retromer complex, mediating the recycling pathway at the TGN and at the MVB^76^. Additionally, our proteomics data identified eight ESCRT proteins (four from ESCRT-0, three from ESCRT-III and one from the SKD1 complex) quantified over all three development stages. MVBs have been suggested to arise by TGN maturation^77,78^ with the support of Rab GTPases^79^. It is worth mentioning that several Rab GTPases were detected over all three different development stages, possibly supporting MVB maturation.

Additionally, ESCRT are known to be necessary to drive the formation of ILVs in MVBs^46–48^. Only few studies concerning ESCRT proteins are described in cereals: in maize, supernumerary aleurone layer1 (Sal1), that encodes the maize homolog of VPS46/CHMP1 (ESCRT-III associated), restricts aleurone cell identity to the outer cell layer of endosperm^80^. SAL1 preserves the proper plasma membrane concentration of DEFECTIVE KERNEL1 (DEK1) and CRINKLY4 (CR4), both involved in aleurone cell fate specification, by regulating the internalization and degradation of SAL1 positive endosomes^74^. The rice AAA ATPase LRD6-6, which is homologous to the AAA ATPase VPS4/SKD1, was identified as an interactor with OsSNF7b/c (Os06g40620/Os12g02830) and OsVPS2.2 (Os03g43860), supporting its putative MVB-mediated vesicular trafficking function^81^. OsLRD6-6 is described to be localized at MVBs, to be required for MVBs-mediated vesicular trafficking and to inhibit the biosynthesis of antimicrobial compounds for the immune response in rice^81^.

Recently, the overexpressed ESCRT-III-associated component HvVPS60 was shown to be involved in protein targeting in developing barley endosperm^12^. Here, seven of the identified ESCRT proteins were most expressed at development phase I and II, indicating a possible involvement in MVB body formation. The high abundances of the ESCRT-0 proteins, HvTOL1, HVTOL2 and HvTOL8 in the development stage I indicate early steps of cargo endocytic events to the vacuole: originally, nine Tom1 (target of Myb1) proteins with a domain structure similar to the VHS domain of ESCRT-0 were identified and it was speculated that these proteins are responsible to load the ESCRT machinery^82^. Tom1 proteins were further characterized as members of the *A. thaliana* TOL family (TOM1-LIKE), which are able to bind ubiquitin directly and participate in the endocytic trafficking of plasma membrane proteins, such as the auxin efflux facilitator PIN2^37^. HvTOL3, which was detected at development phase II, may have an additional/different function, as it was already previously speculated that AtTOLs can participate in different pathways of distinct endosomal systems of plants^83^.

Our proteomics, RT-qPCR, microscopic and biochemical analyses showed a highly similar protein expression and transcription behaviour of both HvSNF7.1/2, which is reasonable as interaction studies showed heterodimerization^54^. Snf7/VPS32 induce membrane curvature at MVBs by assembling into long spiral filaments and are discussed to be involved in corralling the ESCRT cargo at the vesicle bud^84–86^. Given the transgenic line p6U::SNF7.1-mEosFP, which showed punctate structures and are most probably labelling MVBs, and the TEM analyses, we were able to detect MVBs around PBs at early barley endosperm development stages, and later within fused PBs.

To conclude, our proteomic approach combined with *in situ* microscopy, which focused on endomembrane-associated proteins isolated from four different stages of barley grain development, provide only a snapshot of the endomembrane system dynamics. Future studies will be needed to verify the temporal protein-protein interactions identified by the STRING analysis. Furthermore, it would be of interest to study the endomembrane system remodeling under abiotic stress conditions that are likely to affect the SSPs synthesis and/or the production of recombinant proteins. Finally, experimental proof of the involvement of cytoskeleton-related proteins, MVBs and ESCRT in sorting proteins to the PBs, ideally obtained by investigating mutant barley lines impaired in ESCRT function using proteomics and *in situ* microscopy, has yet to be provided. Nevertheless, the proteins that have been identified in association with the endomembrane system are useful targets for genetic engineering to modulate SSPs accumulation.

## Supplementary information

Supplementary information.

Supplemental table 1.

Supplemental table 2.
