# Supplementary material for: Protein sorting into protein bodies during barley endosperm development is putatively regulated by cytoskeleton members, MVBs and the HvSNF7s
Source: Sci Rep. 2020 Feb 5;10:1864. doi: 10.1038/s41598-020-58740-x (PMC7002727; doi:10.1038/s41598-020-58740-x)
Supplement: Supplementary file 1 — Supplementary information. [file 41598_2020_58740_MOESM1_ESM.pdf]

Protein sorting into protein bodies during barley endosperm development is putatively regulated by cytoskeleton members, MVBs and the HvSNF7s

Valentin Roustan[1], Julia Hilscher[2], Marieluise Weidinger[3], Siegfried Reipert[3], Azita Shabrangy[1], Claudia Gebert[1], Bianca Dietrich[2#], Georgi Dermendjiev[1], Madeleine Schnurer[1], Pierre-Jean Roustan[1], Eva Stoger[2] and Verena Ibl[1*]

[1]Department of Ecogenomics and Systems Biology, University of Vienna, Althanstr. 14, 1090 Vienna, Austria

[2]Department for Applied Genetics and Cell Biology, University of Natural Resources and Life Sciences, Muthgasse 18, 1190 Vienna, Austria

[3]Core Facility Cell Imaging and Ultrastructure Research, University of Vienna, Althanstr. 14, 1090 Vienna, Austria

[#]current address: Medical University of Vienna, Department of Obstetrics and Gynecology

Reproductive Biology Unit, Währinger Gürtel 18-20, 5Q, A-1090 Vienna, Austria

*Correspondence: Dr. Verena Ibl, Department of Ecogenomics and Systems Biology, University of Vienna, Althanstr. 14, 1090 Vienna, Austria, verena.ibl@univie.ac.at , Twitter: @VerenaIbl

a

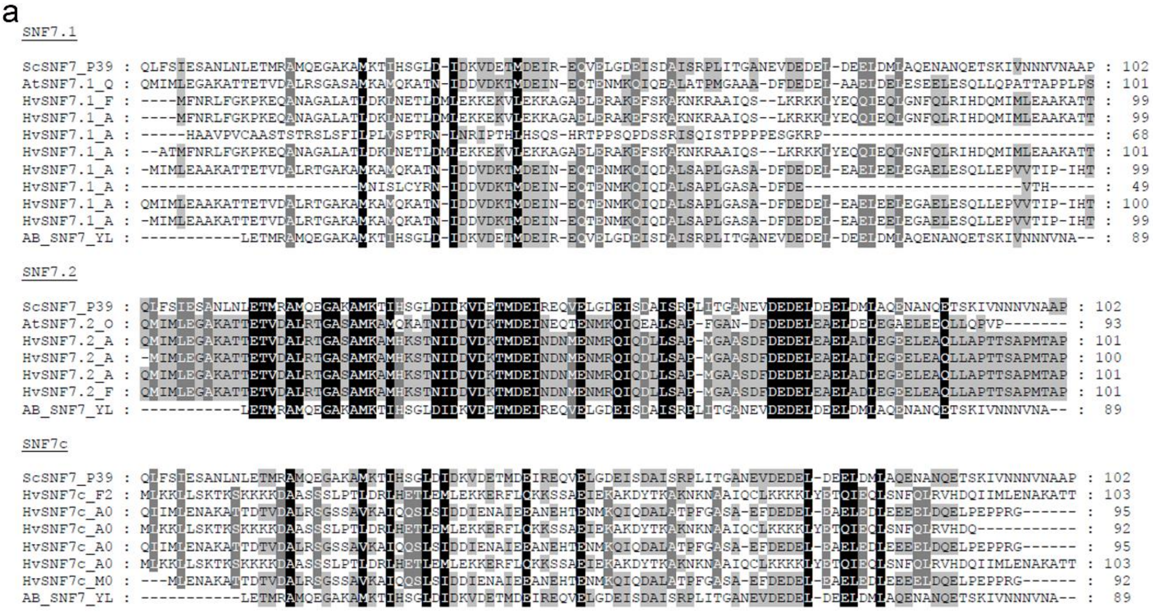

b

Name	Gene	full length cDNA	UniProt	AA length	kDA	SNF7 antibody recognition
SNF7.1/B	HORVU5Hr1G052710.6	AK358256	F2D2Z9	220	24.35	+
			**A0A287R803**	119	13.45	+
			A0A287R8G4	68	68	+
			A0A287R882	222	24.56	+
			A0A287R826	123	13.28	++
			A0A287R8G9	49	5.53	++
			A0A287R8D8	155	17.14	++
			A0A287R876	132	14.18	++
SNF7.2/A	HORVU7Hr1G090760.2		**A0A287XAB9**	284	31.38	+++
			A0A287XAF0	129	13.9	+++
			A0A287XAE1	178	19.773	+++
		AK354835	F2CT83	222	24.49	+++
SNF7c	HORVU5Hr1G045960.3	AK366149	F2DQI1	218	24.28	++
			A0A287R1E9	159	17.47	++
			A0A287R1Q5	96	11.21	++
			A0A287R190	190	21.12	++
			A0A287R1H9	149	17.17	++
			M0V3N1	124	13.32	++

d

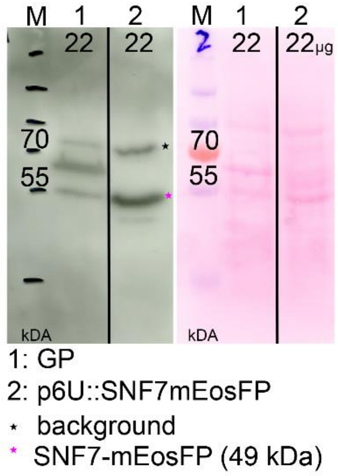

c

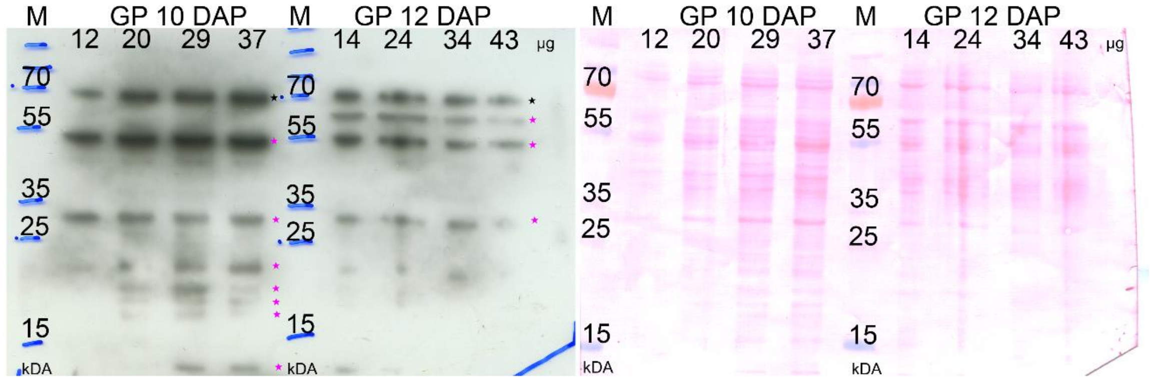

Figure S1. Western blot of barley wild-type (GP) and transgenic line p6U::SNF7.1-mEosFP. (**a**) Alignment of *Saccharomyces cerevisiae (Sc)* ScSNF7, HvSNF7.1, HvSNF7.2 and HvSNF7c and the sequence of the polyclonal rabbit anti-ScSNF7 [1]. Note the common amino acids. For *Arabidopsis* following gene and protein codes were used: AtSNF7.1: At4g29160, Uniprot: Q9SZE4; AtSNF7.2: At2g19830, Uniprot: O82197; for ScSNF7: Uniprot: P39929; for *Hordeum vulgare* note the table in (b). (**b**). Table of all *Hordeum vulgare* HvSNF7 isoforms including information of the gene, full length cDNA, amino acid (AA) length, protein size (kDa) and the possibility of the SNF7 antibody recognition according to the number of identic AAs. (**c**) Detection of HvSNF7.1, HvSNF7.2 and HvSNF7c

of total protein extracts of GP grains at 10 and 20 DAP using the polyclonal rabbit anti-SNF7. black asterisks – background band, magenta asterisks – positive signals from hetero- homodimers and different isoforms of HvSNF7. The amount of loaded sample is indicated in µg. Note the stage dependent signal of 10 and 12 DAP. Exposure time of the film was 10 minutes to visualize the weak bands below 25 kDa. After film development, the membrane was stained with Ponceau S. (**d**) Polyclonal rabbit anti-SNF7 confirms the intact fusion protein p6U::SNF7.1-mEosFP of total proteins extracts of this transgenic line. black asterisk – background band, magenta asterisk – positive signals from p6U::SNF7.1-mEosFP (49 kDa). In total, 22 µg protein was loaded. Note the strong signal from p6U::SNF7.1-mEosFP compared to the signal of GP. Exposure time of the film was 3 minutes. After film development, the membrane was stained with Ponceau S. Western blot is grouped of cropped lines from the same blot, indicated by the dividing lines.

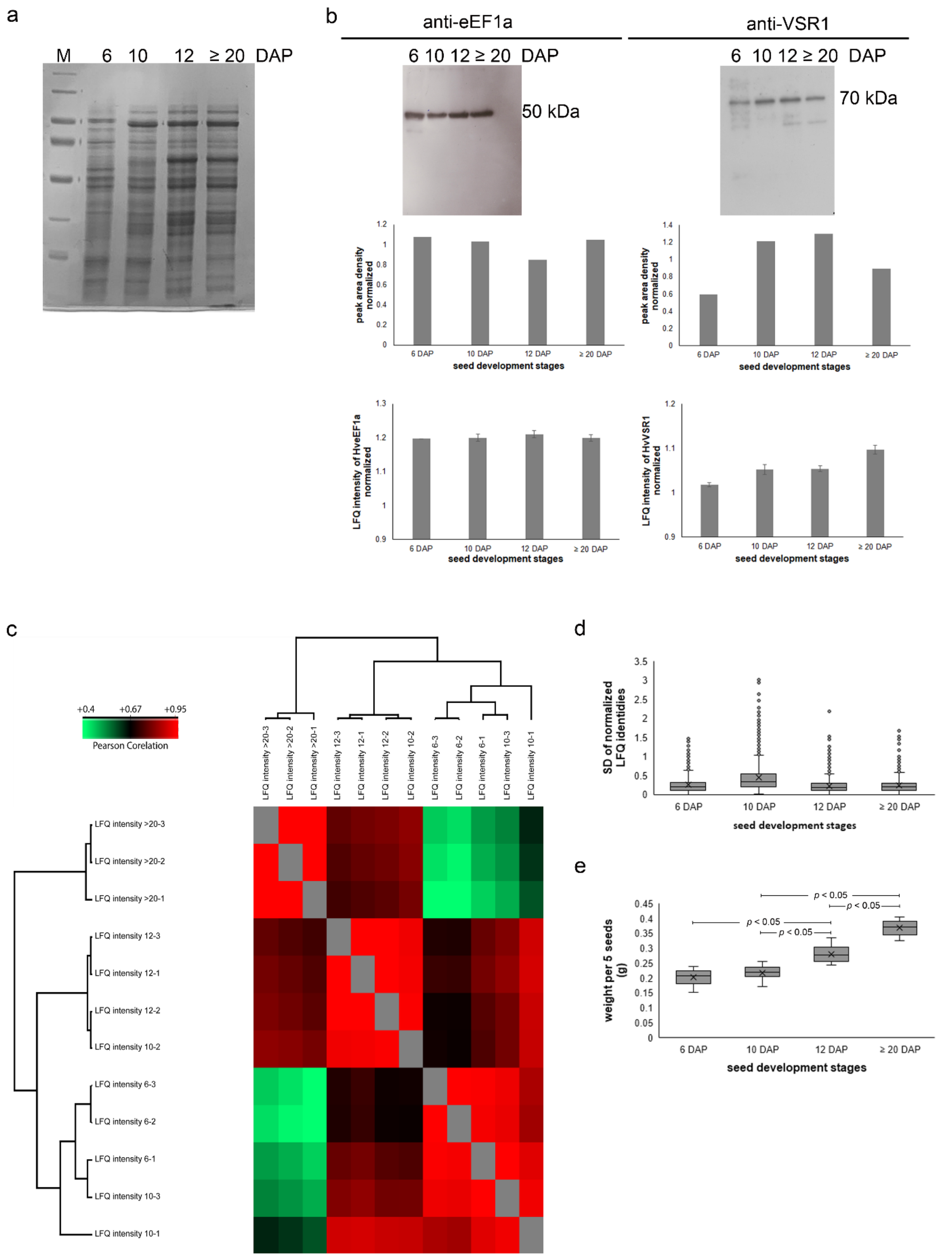

Figure S2. SDS-Page, Western blot and PCA confirm the consistency of the experimental set-up and of the obtained data. (**a**) SDS-PAGE of total protein extracts of GP grains at 6, 10, 12 and ≥ 20 DAP. (**b**)

Semi-quantitative Western blot analyses with anti-eEF1a and anti-VSR1 of protein extracts of different development stages reveal a constant and an increasing signal, respectively. The signal intensities (peak area density) of the Western blots were quantified by ImageJ. Normalized LFQ intensities of identified HveEF1a (F2EG53) and HvVSR1 peptides (A0A287H7X6, A0A287NZS5, A0A287R1U7) at 6, 10, 12 and ≥ 20 DAP were averaged over three biological replicates. Bars represent standard deviation. (**c**) Hierarchical-Clustering-Analysis shows that the measured protein abundances were highly reproducible with an average Pearson's correlation coefficients of 0.96 between biological replicates. (**d**) Standard deviation of the normalized LFQ intensities between 6 and ≥ 20 DAP. (**e**) Seed weight per 5 seeds (g) averaged of 3 biological replicates at different development stages.

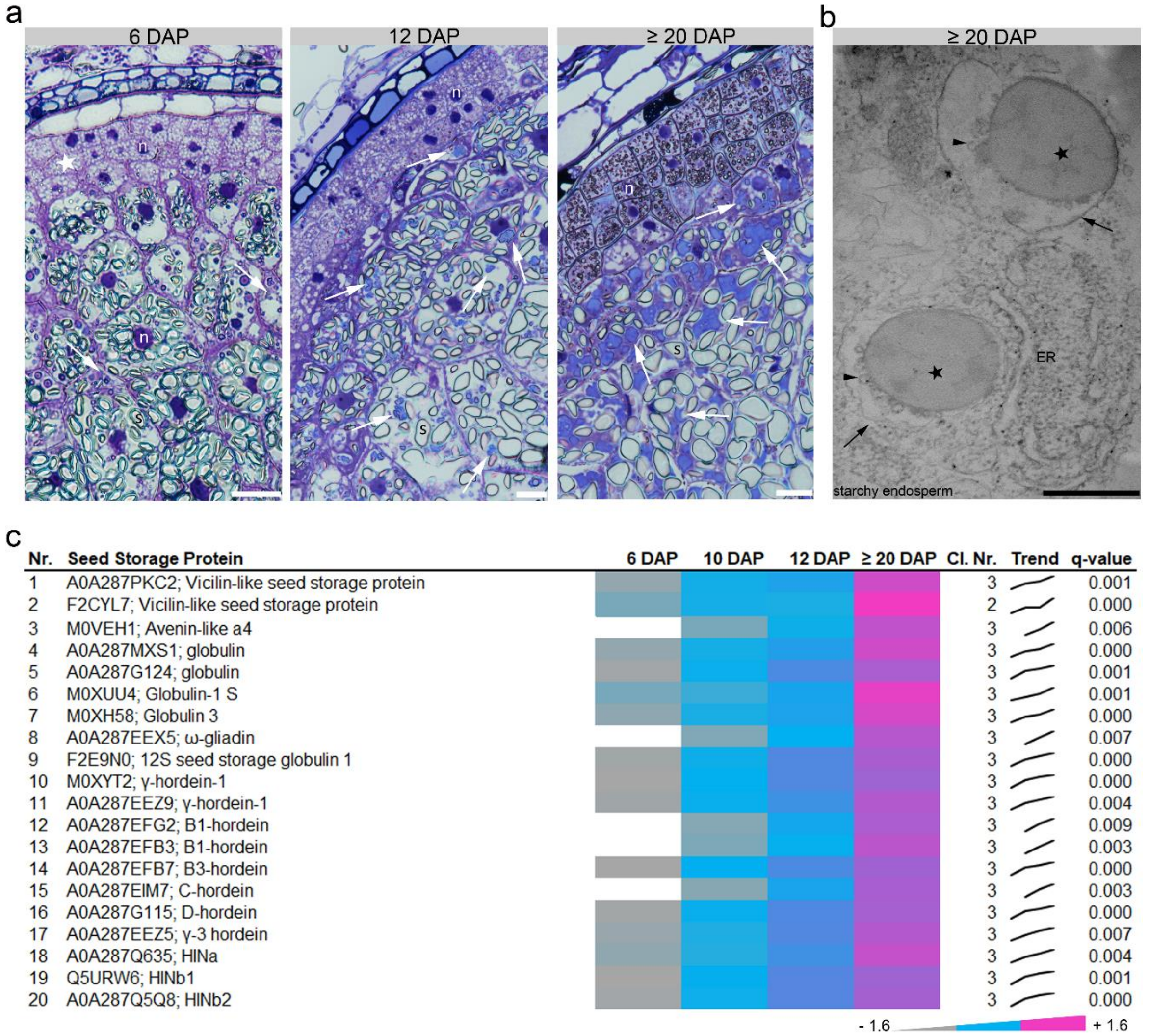

Figure S3. Cellularization and identification of SSPs in developing barley grains. (**a**) Toluidine blue staining of sections prepared at 6, 12 and ≥ 20 DAP. At 6 DAP, cellularization of the barley endosperm is finished and three aleurone cell layers are shown. Toluidine blue-stained compartments (arrows) were more abundant at ≥ 20 DAP compared to 12 and 6 DAP, confirming that the protein level of SSPs increase during development. Note the indicated tissues: aleurone, subaleurone and starchy endosperm. Bars = 100 µm. (**b**) Single, spherical PBs (asterisks) were observed by TEM in starchy endosperm cells engulfed by putative vacuolar membranes (arrows). Note vesicles (arrowheads) attached to the PBs. ER, endoplasmic reticulum. Bar = 0.5 µm. (**c**) Data-matrix heat map representing z-score values of 6, 10, 12 and ≥ 20 DAP. Heat map was prepared using Microsoft Excel. Scale: grey = smallest value; blue = 50% quantile; pink = highest value. Identified proteins involved in the accumulation of SSPs were highest at ≥ 20 DAP. Note that all identified SSPs could be found in cluster 3 except F2CYL7; Vicilin-like seed storage protein, that was grouped into cluster 2.

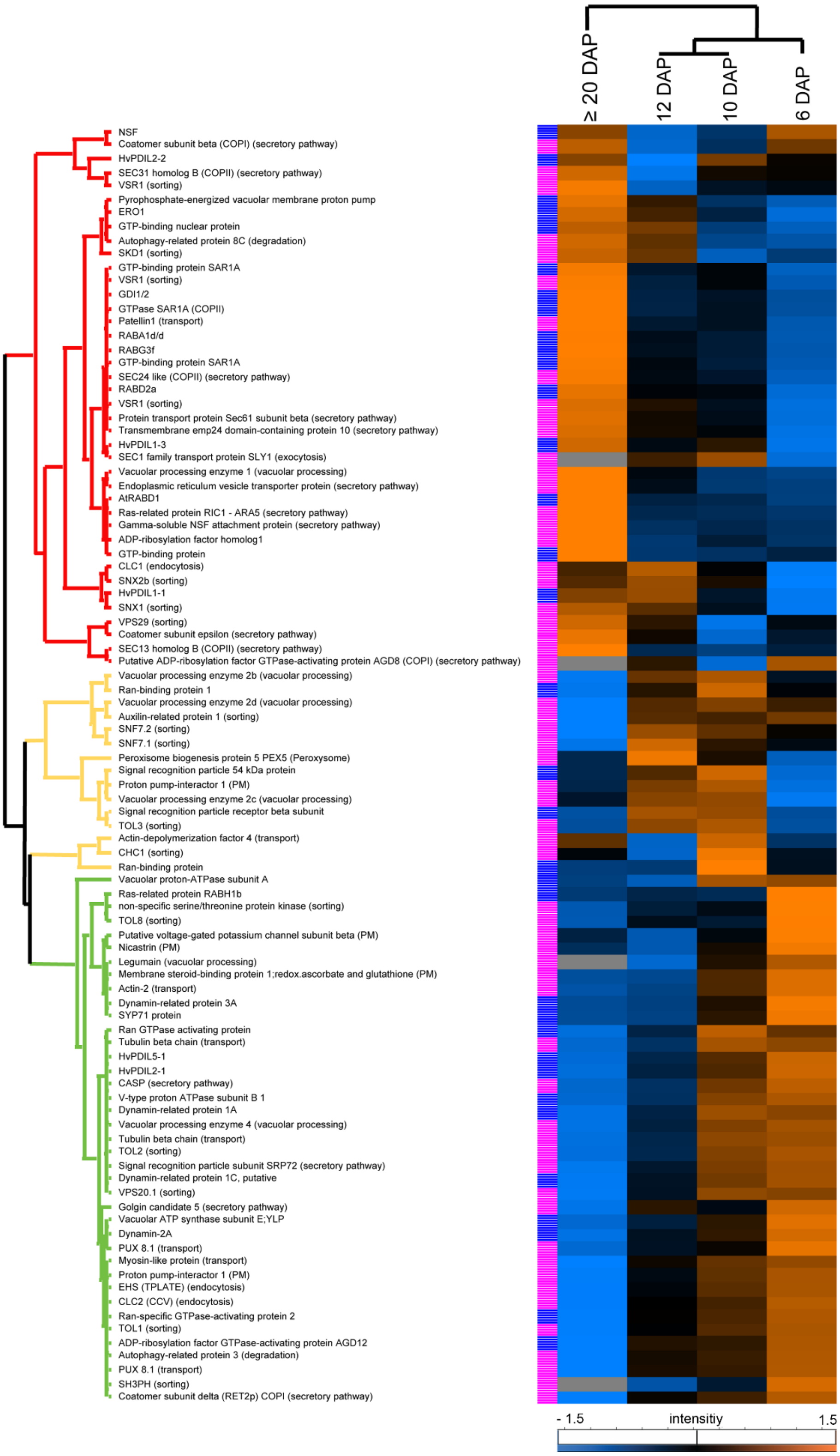

Figure S4. Heat map visualizes the protein expression pattern based on Pearson correlation at 6, 10, 12 and ≥ 20 DAP and clusters the proteins into three main groups. 39 proteins present a higher expression during the development phase I (green); 15 proteins presented an expression peak at development phase II (yellow); 40 proteins, associated with development phase III (red). Compartment-specific proteins and trafficking regulators are indicated with pink and blue, respectively.

a

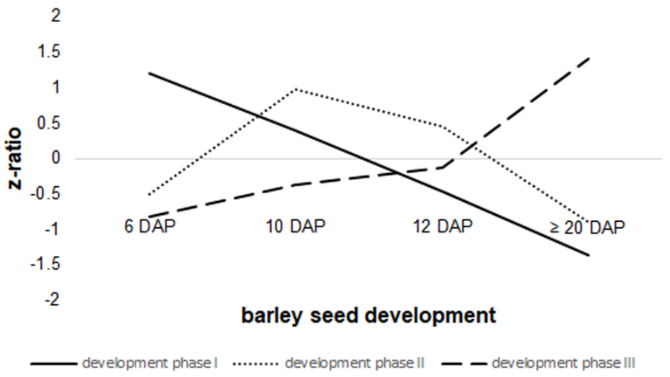

b

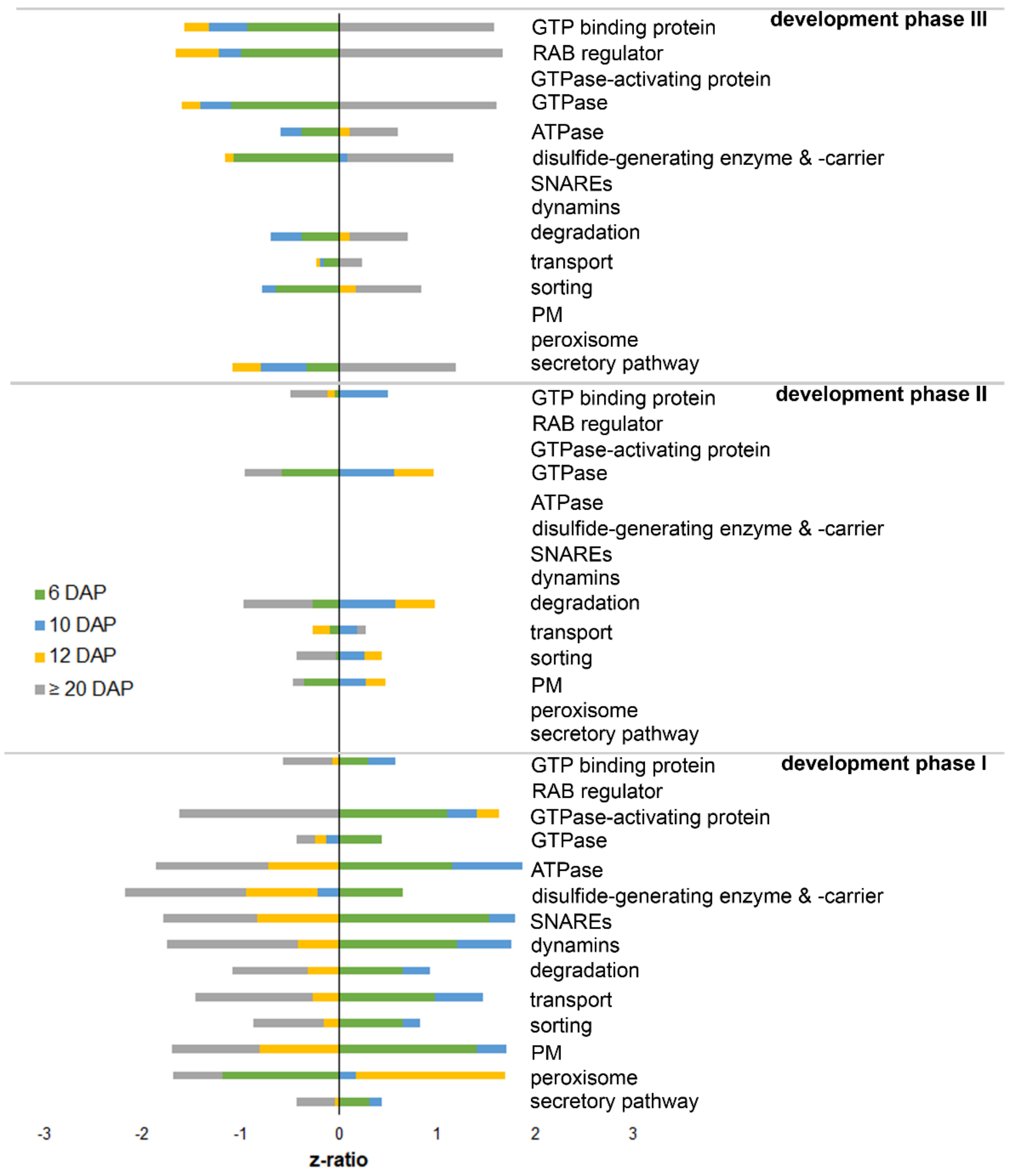

Figure S5. Proteins associated to the endomembrane system are clustering into three different development phases. (**a**) Mean of z-score values of all proteins identified in the different development phases visualizing that development phase I, II, and III behave differently during endosperm development. (**b**) Mean of z-score values of spatio- functional active proteins within development phase I, II and III.

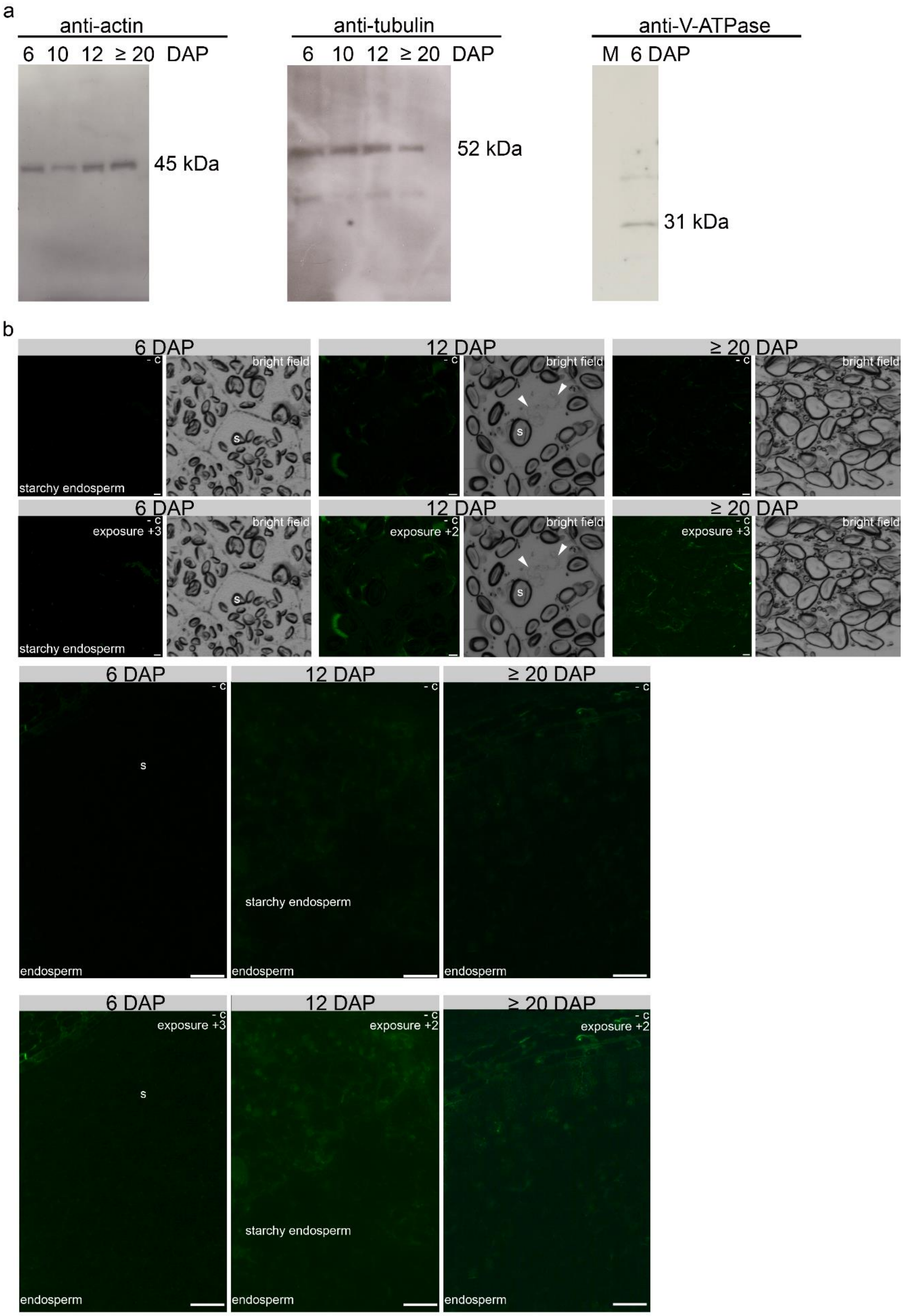

Figure S6. Immunofluorescence of sections prepared from 6, 12 and ≥ 20 DAP. (**a**) Western blot analyses with anti-actin, anti-tubulin and anti-V-ATPase of protein extracts of different development stages reveals specific signals, respectively. 6 DAP was chosen to proof the already commercially confirmed specificity of anti-V-ATPase in barley. (**b**) Negative controls from the anti-actin and anti-tubulin-α immunofluorescence assay from section prepared of 6, 12 and ≥ 20 DAP

analyzed by confocal microscopy. Negative controls show sections incubated only with the secondary antibody. Pictures are included with an extended exposure time (two/three times longer) to visualize the sections (indicated by exposure +2/3). Additionally, Corresponding negative controls from section prepared of 6, 12 and ≥ 20 DAP analysed by fluorescence microscopy. Negative controls show sections incubated only with the secondary antibody. Pictures are included with an extended exposure time (two/three times longer) to visualize the sections (indicated by exposure +2/3). M: Thermo Scientific PageRuler prestained protein ladder (10 bis 180 kDa), bar = 100 µm.

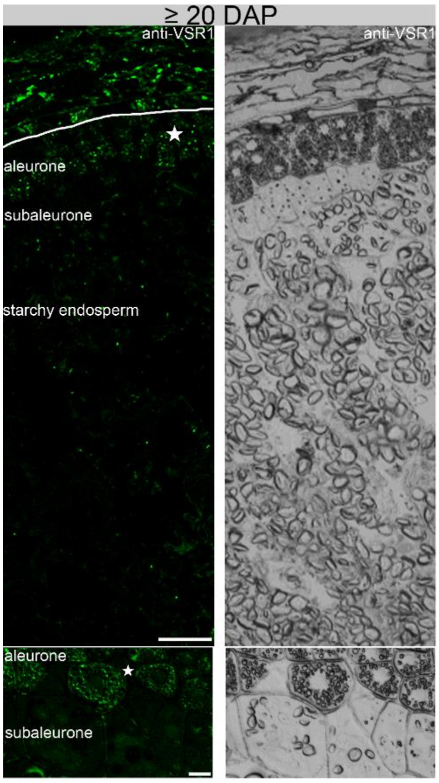

Figure S7. VSR1 localization. Sections prepared at ≥ 20 DAP were incubated with anti-VSR1 and analyzed by confocal microscopy. Asterisks show the strong signal in the aleurone layer. Note the zoom at the bottom. Bar = 50 µm and 10 µm, respectively.

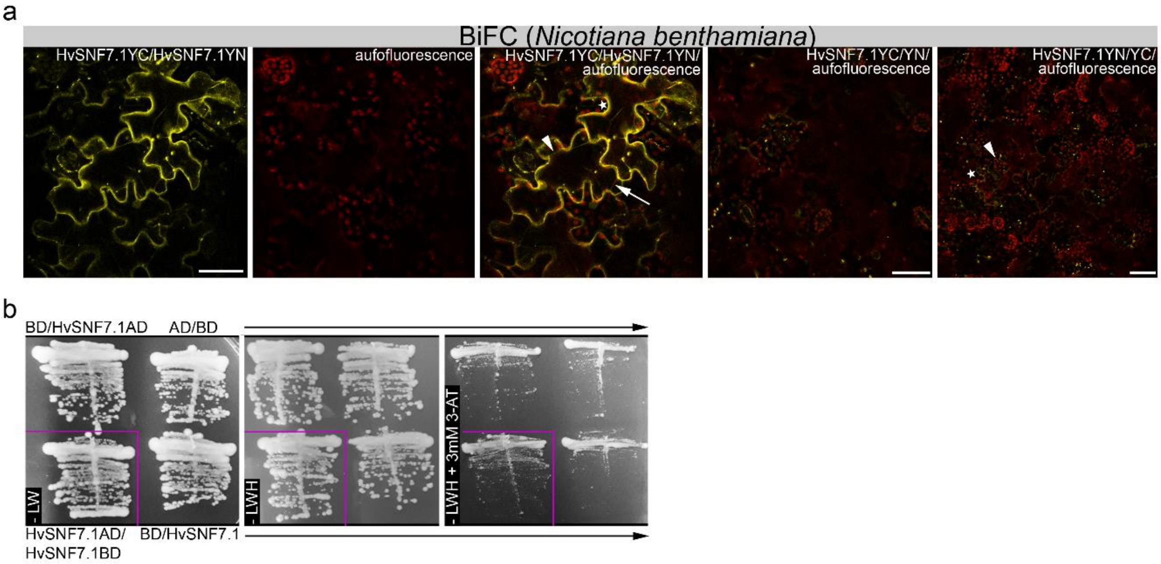

Figure S8. Homodimerization of HvSNF7.1. Homodimerization of SNF7.1 shown by BiFC (**a**) and Y2H (**b**).

Table S1. Selected RGs are listed by their gene number, gene symbol, gene product function, gene ID (Unigene), primer sequence, amplicon length, corresponding mRNA sequence, corresponding protein (Uniprot) entry, and the identified protein in our MS data.

Tabel S2: MS data. Table S2a: Protein Group quantification and annotation; Table S2b: Loading of the PCA; Table S2c: Results of GProX fuzzy-mean clustering analysis.

Materials and methods

Sample preparation for proteomics analyses

Total proteins were extracted from approximately 20 barley grains (one ear) harvested at 6-8, 10, 12-18 and ≥ 20 DAP in three biological replicates. Stages were chosen as previously described [2]. Barley flowers were not emasculated, and DAP were counted from the first dehiscence of anthers. Extraction was performed following an adapted phenol-phase extraction protocol as described in [3]. Subsequently, proteins were re-suspended in urea buffer (8 M urea, 100 mM ammonium bicarbonate) to measure protein concentration with a Bradford Assay prior protein content normalization and trypsin digestion. Following overnight digestion, peptides were desalted using a C18 solid phase extraction (Agilent Technologies, Santa Clara, USA). After solid-phase extraction, the corresponding eluates were dried in a vacuum concentrator.

Nano-HPLC and Orbitrap Elite tune methods

Mass spectrometry (MS) was performed as previously described [4]. Peptide pellets were resolved at a protein concentration equivalent of 0.1 µg/µL. A total of 0.5 µg of the mixture was separated on an EASY-Spray PepMap RSLC 75 µm × 50 cm column (Thermo Fisher Scientific, Waltham, Massachusetts, USA). After elution using a 150 min linear gradient at a 300 nL/min flow rate generated with an UltiMate 3000 RSLCnano system, the peptides were measured with an LTQ-Orbitrap Elite (Thermo Fisher Scientific, Waltham, Massachusetts, USA) as described in [4]. Mass analyzer settings were as follows: ion transfer capillary temperature 275 °C, full scan range 350–1800 m/z, FTMS resolution 60,000. Each FTMS full scan was followed by up to twenty data-dependent (DDA) CID tandem mass spectra (MS/MS spectra) in the linear triple quadrupole (LTQ) mass analyzer. Dynamic exclusion was enabled using list size 500 m/z values with an exclusion width ±10 ppm for 60 s. Charge state screening was enabled and unassigned and +1 charged ions were excluded from MS/MS acquisitions. For injection control, automatic gain control for full scan acquisition in the Orbitrap was set to 1 x 106 ion population, and the maximum injection time (max IT) was set to 200 ms. Multistage activation was enabled with neural losses of 24.49, 32.66, 48.999, 97.97, 195.94, and 293.91 Da for the 10 most intense precursor ions. Prediction of ion injection time was enabled, and the trap was set to gather 5 x 103 ions for up to 100 ms. Orbitrap online calibration using internal lock mass calibration on m/z 371.10123 from polydimethylcyclosiloxane was used.

Western blots

To confirm the MS results, we have used the same extraction protocol for SDS Page and the Western blots as for the MS analysis. For SDS page, between five and seven wild type (GP) seeds of 6 and 10 DAP, and three seeds of 10, 12 and ≥ 20 DAP were taken for protein extraction. After protein extraction, we measured the quantity of protein with a Bradford assay. 15 ug total protein amount were loaded of each sample and the gel was stained for 30 min by Coomassie and destained overnight. A total of 2 µg of PageRuler Plus Prestained Protein Ladder (#26 619, Thermo Fisher Scientific, Waltham, Massachusetts, USA) was loaded on a 10% acrylamide gel. For the analysis of the transgenic line p6U::SNF7.1-mEosFP, five transgenic and wild type (GP) grains were extracted by PBS buffer pH 6. After protein extraction, we measured the quantity of protein with a Bradford assay. Western blots were performed as previously described [4]. For the analyses of the specificity of anti-V-ATPase, proteins were extracted at 6 DAP as described in [2] and 4 µl was loaded. A total of 2 µg of PageRuler Plus Prestained Protein Ladder (#26 619, Thermo Fisher Scientific, Waltham, Massachusetts, USA) and 20 µg of the samples were loaded on a 10% acrylamide gel. Antibodies were diluted as following: polyclonal rabbit anti-V-ATPase antibody (#AS 07 213, Agrisera, Vännäs, Sweden), dilution 1:5000; actin (#AS13 2640, Agrisera, Vännäs, Sweden), 1:5000; tubulin alpha (#AS10 680, Agrisera, Vännäs, Sweden), 1:1000 polyclonal rabbit anti-SNF7 antibody (kindly provided by [1], 1/1000; and Amersham ECL Rabbit IgG, HRP-linked whole Ab (from donkey) (#NA934VS, GE Healthcare, Illinois, Chicago, United

States), 1/10,000. The ECL prime Western blotting detection reagent (#RPN2232, GE Healthcare, Illinois, Chicago, United States) was used for development.

Phenotyping of barley grains

The weight was measured each time of five GP seeds of three biological replicates at 6, 10, 12 and ≥ 20 DAP. Data was visualized by a boxplot.

RT-qPCR Analysis

RT-qPCR analysis of *ESCRTs* was performed according to the MIQE guidelines [5]. RNA was isolated from grains at four development stages: 6, 10, 12 and ≥ 20 DAP (RNeasy PowerPlant #13500-50, Qiagen, Netherlands). The RNA concentration was measured at 260 nm using a UV spectrophotometer (NanoDrop Technologies, Thermo Fisher Scientific, Massachusetts, USA) and ranged from 70 to 224 ng/µL. RNA integrity was assessed by microfluidic capillary gel electrophoresis using the ExperionTM RNA HighSens Analysis Kit (#7007105, Bio-Rad Laboratories, Hercules, Carlifornia, USA) with Experion software 3.2P. The quality of RNA index (QRI) showed acceptable quality of the isolated RNA. Next, cDNA was synthesized as recently described in [2]. Specific primers were designed for *HvPDIL1-1* using Primer-Blast (https://www.ncbi.nlm.nih.gov/tools/primer-blast/) (see Supplemental Table 1).

Besides bioinformatic analyses, the specificity of the primers was tested by PCR on the cDNAs. At least three biological replicates were used, and three technical replicates were performed for RT-qPCR. For the normalization studies of *ESCRT* transcripts, we used the following reference genes as described in [2]: *ARF* (ADP-ribosylation factor), *FBPA* (fructose-bisphosphate aldolase), and *SAM* (S-adenosyl-L-methionine) for whole grains. Normalization was calculated as described in [2,6]. For statistical analyses we performed a Student's t-test (two-tailed distribution, two-sample unequal variance (heteroscedastic)) using the Microsoft Excel software program.

Yeast two-hybrid (Y2H)

The vector pGBKT7-HvSNF7.1 was co-transformed with pGADT7-HvSNF7.1 into the yeast strain Y190. In addition, pGBKT7-HvSNF7.1 was co-transformed with pGBKT7, pGBKT7 with pGADT7-HvSNF7.1 and pGBKT7 with pGADT7. Transformation was performed as following: a 3mL preculture (YPD media, yeast extract 5 g, tryptone 10 g, glucose 10 g) was prepared from a single colony of Y190 and incubated overnight at 30 °C. About 10–15 mL of YPD media were inoculated with 1 ml preculture and incubated at 30 °C for 3 h. Cells were accumulated by centrifugation at 3,000 rpm at 20 °C for 5 min. Cells were washed with 1 mL TE buffer (10 mM Tris/HCL pH 7.5, 1mM EDTA pH 8.0) and re-centrifuged at 3,000 rpm at 20 °C for 5 min. LA-buffer (Li-Acetat*2H_2O 1.02 g, in 100 ml TE-buffer pH 7.5) was mixed with the yeast cells and 60 µg of DNA was added. Transformation was performed adding 300 µL 50% PEG4000 (#0156.3, Carl Roth, Karlsruhe, Germany), incubated at 22 °C for 1 h, then adding 40 µL 100% DMSO and incubating at 42 °C for 10 min. Cells were collected by centrifugation and washed with TE buffer. Transformants were selected after 2 d on synthetic dropout (SD) medium lacking Leu and Trp (SD-LW) at 30 °C. To examine Y2H interactions, the transformants were streaked out on solid medium lacking Leu and Trp (SC-LW) or lacking Leu, Trp, and His (-LWH) with 5 mM 3-amino-1,2,4-triazole for 2 days at 30 °C.

References

1 Teis, D., Saksena, S., Judson, B. L. & Emr, S. D. ESCRT-II coordinates the assembly of ESCRT-III filaments for cargo sorting and multivesicular body vesicle formation. *The EMBO journal* **29**, 871-883, doi:10.1038/emboj.2009.408 (2010).

2 Shabrangy, A. *et al.* Using RT-qPCR, Proteomics, and Microscopy to Unravel the Spatio-Temporal Expression and Subcellular Localization of Hordoindolines Across Development in Barley Endosperm. *Frontiers in Plant Science* **9**, doi:doi: 10.3389/fpls.2018.00775 (2018).

3 Roustan, V., Bakhtiari, S., Roustan, P.-J. & Weckwerth, W. Quantitative in vivo phosphoproteomics reveals reversible signaling processes during nitrogen starvation and recovery in the biofuel model organism Chlamydomonas reinhardtii. *Biotechnology for biofuels* **10**, 280, doi:10.1186/s13068-017-0949-z (2017).

4 Roustan, V. *et al.* Microscopic and Proteomic Analysis of Dissected Developing Barley Endosperm Layers Reveals the Starchy Endosperm as Prominent Storage Tissue for ER-Derived Hordeins Alongside the Accumulation of Barley Protein Disulfide Isomerase (HvPDIL1-1). *Front Plant Sci*, doi:https://doi.org/10.3389/fpls.2018.01248 (2018).

5 Bustin, S. A. *et al.* The MIQE Guidelines: Minimum Information for Publication of Quantitative Real-Time PCR Experiments. *Clinical Chemistry* **55**, 611-622, doi:10.1373/clinchem.2008.112797 (2009).

6 Vandesompele, J. *et al.* Accurate normalization of real-time quantitative RT-PCR data by geometric averaging of multiple internal control genes. *Genome Biol* **3**, RESEARCH0034 (2002).
